# Supplementary figures and images for: Characteristics, demographics, and epidemiology of possible chronic cough in Sweden: A nationwide register-based cohort study
Source: PLoS One. 2024 Jul 24;19(7):e0303804. doi: 10.1371/journal.pone.0303804 (PMC11268580; doi:10.1371/journal.pone.0303804)

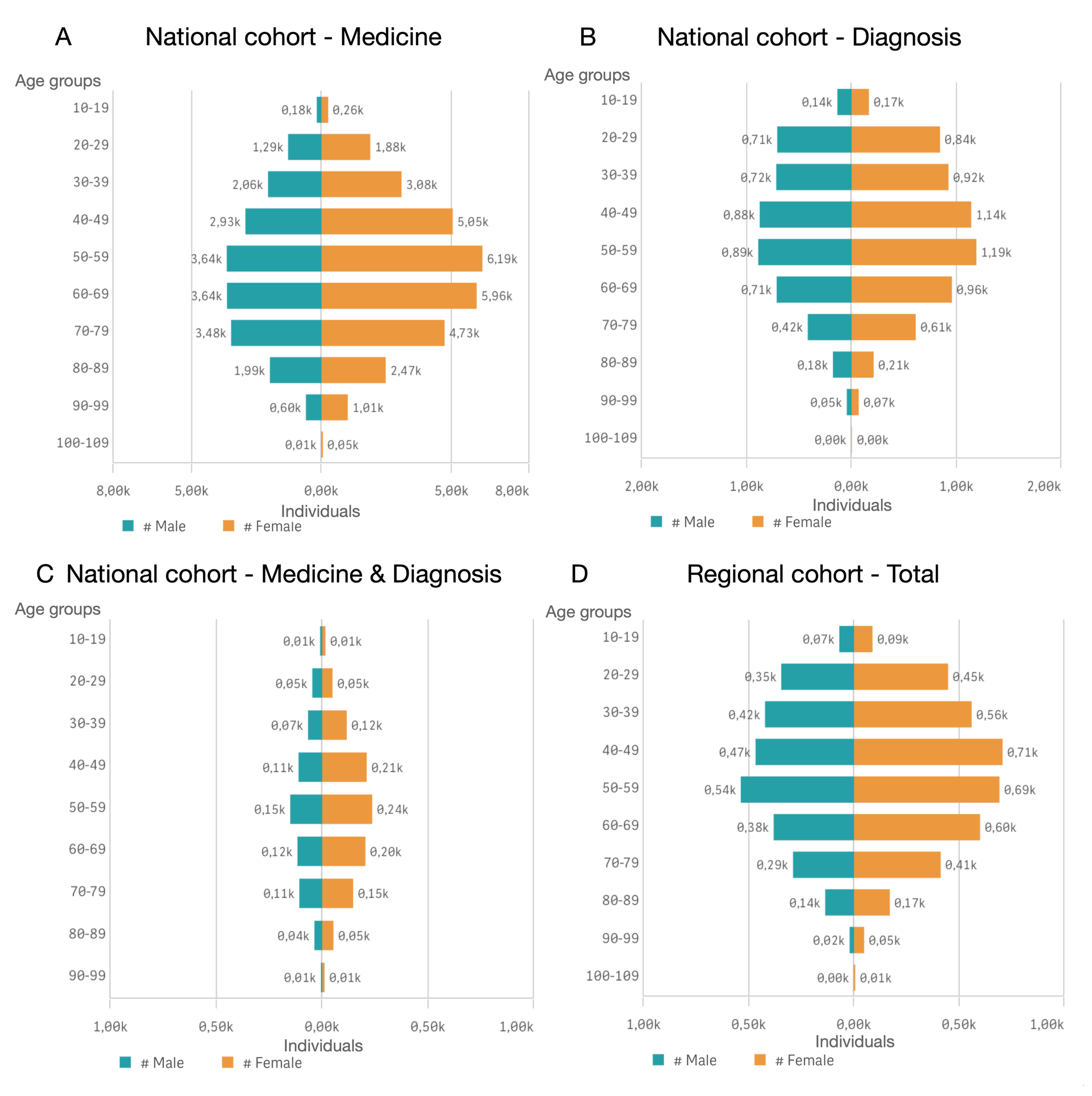

Supplement: S1 Fig — (TIF) [file pone.0303804.s001.tif]

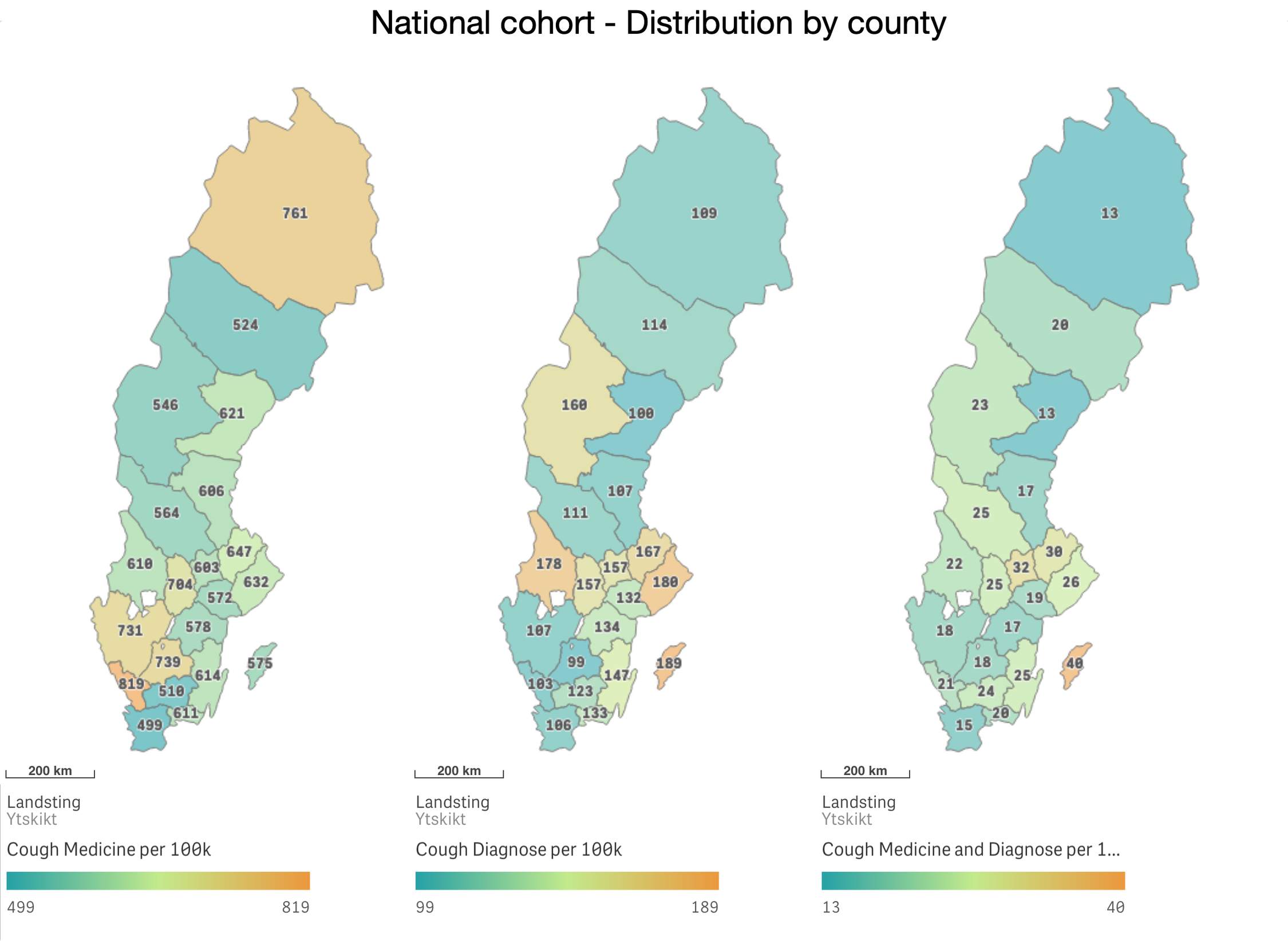

Supplement: S2 Fig — (TIF) [file pone.0303804.s002.tif]
