# Supplementary material for: Characteristics, demographics, and epidemiology of possible chronic cough in Sweden: A nationwide register-based cohort study
Source: PLoS One. 2024 Jul 24;19(7):e0303804. doi: 10.1371/journal.pone.0303804 (PMC11268580; doi:10.1371/journal.pone.0303804)
Supplement: S1 File — (DOCX) [file pone.0303804.s003.docx]

**Supplement Tables**

**Table A**. Number of individuals with possible chronic cough stratified by type of cough medicine and number of dispenses per cough medicines 2016-2018.

| Group | ATC | Name | 1  DISP | 2-3  DISP | 4-6  DISP | >6  DISP | Total |
| --- | --- | --- | --- | --- | --- | --- | --- |
| Exp | R05CB01 | Acetylcysteine | 6,440  20.7 % | 12,090  38.9 % | 7,220  23.2 % | 5,301  17.1 % | 31,051  100.0 % |
| Opiates | R05DA04 | Codeine | 314  17.2 % | 484  26.5 % | 325  17.8 % | 705  38.6 % | 1,828  100.0 % |
| CS/ Exp | R05CB02 | Bromhexine | 225  40.8 % | 131  23.7 % | 75  13.6 % | 121  21.9 % | 552  100.0 % |
| CS/ Exp | R05FB02 | Cough Suppressants and expectorants | 162  47.5 % | 122  35.8 % | 44  12.9 % | 13  3.8 % | 341  100.0 % |
| CS | R05DA07 | Noscapine | 220  80.6 % | 48  17.6 % | 4  1.5 % | 1  0.4 % | 273  100.0 % |
| CS | R05DA20 | Combinations | 1,269  49.8 % | 964  37.8 % | 255  10.0 % | 60  2.4 % | 2,548  100.0 % |
| CS | R05FA02 | Opium derivatives and expectorants | 8,868  25.8 % | 19,224  56.0 % | 5,163  15.0 % | 1,074  3.1 % | 34,329  100.0 % |
| Anti-E | R06AA02 | Dimenhydrinate | 7  36.8 % | 9  47.4 % | 3  15.8 % | 0  0.0 % | 19  100.0 % |
| Total | | | 14,841  26.6 % | 31,454  56.3 % | 12,976  23.2 % | 7,206  12.9 % | 55,823  100.0 % |

One patient may have multiple medicines and be counted on multiple rows. Exp: Expectorants, CS: Cough Suppressant, Anti-E: Anti-emetic.

**Table B.** Individuals identified with cough in the national cohort (25, 046) and the regional cohort (11,647). Stratification of excluded patients.

| Group | Time | Code | Excluded by | | Total |
| --- | --- | --- | --- | --- | --- |
|  |  |  | **National cohort** | **Regional cohort** |  |
| ACEi | Anytime | ATC: C09 | 106,675 | 2,533 | 109,208 |
| Antibiotics | +/- 8w from ID | ATC: J01 | 140,250 | 2,331 | 142,637 |
| Cancer | Anytime | ICD: C32, C33 and C34 | 4,374 | 39 | 4 413 |
| Acute cough | +/- 8w from ID | ICD: J06, J20, J21 and J22 | 10,255 | 2,027 | 12,599 |
| Pneumonia | +/- 8w from ID | ICD: J15, J16, J17 and J18 | 21,063 | 728 | 21,804 |
| TBC | Anytime | ICD: A15, A16, A18 and A19 | 256 | 6 | 262 |
| Emergency visit for R05 | Anytime | ICD: R05 | 1,532 | 0 | 1,532 |
| Other lung diseases | Anytime | ICD: J84 | 3,191 | 35 | 3,227 |
| COPD | Anytime | ICD: J44 | 32,217 | 462 | 32,707 |
| Total | | | 193,083 | 5,249 | 198,697 |

One patient might have multiple exclusion criteria fulfilled for the same exclusion step and thus be counted on multiple rows.

**Table C.** Description of the ICD-10 and ATC codes used in exclusion.

| Exclusion group | ATC/ICD | Description |
| --- | --- | --- |
| ACEi | ATC C09 | Agents acting on the Renin-Angiotensin system |
| Antibiotics | ATC J01 | Anti-bacterials for systemic use |
| Cancer | C32 | Malignant Neoplasm Of Larynx |
|  | C33 | Malignant Neoplasm Of Trachea and Bronchus |
|  | C34 | Malignant Neoplasm Of Lung |
| Acute cough | J06 | Acute Upper Respiratory Infections Of Multiple And Unspecified Sites |
|  | J21 | Acute Bronchiolitis |
|  | J22 | Unspecified Acute Lower Respiratory Infection |
| Pneumonia | J15 | Bacterial Pneumonia, Not Elsewhere Classified |
|  | J16 | Pneumonia Due To Other Infectious Organisms, Not Elsewhere Classified |
|  | J17 | Pneumonia In Diseases Classified Elsewhere |
|  | J18 | Pneumonia, Unspecified Organism |
| Tuberculosis | A15 | Respiratory Tuberculosis |
|  | A16 | Respiratory Tuberculosis, Not Confirmed Bacteriologically Or Histologically |
|  | A18 | Tuberculosis Of Other Organs |
|  | A19 | Miliary Tuberculosis |
| Emergency Trtmnt | R05 | Acute or Emergency treatment of cough |
| Other lung diseases | J84 | Other Interstitial Pulmonary Diseases |
| COPD | J44 | Other Chronic Obstructive Pulmonary Disease |
|  |  |  |

**Table D.** Diagnoses used for RCC classification and number of individuals per group.

| Co-diagnosis at R05 visit 2016-2018 | | | | |
| --- | --- | --- | --- | --- |
| Diagnosis | **Individuals** | | | **%** |
| R06 Abnormalities of Breathing | | 737 | 20.7 | |
| J30 Vasomotor and Allergic Rhinitis | | 413 | 11.6 | |
| R50 Fever of Other and Unknown Origin | | 374 | 10.5 | |
| J45 Asthma | | 369 | 10.4 | |
| R07 Pain in Throat and Chest | | 285 | 8.0 | |
| K21 Gastro-Esophageal Reflux Disease | | 246 | 6.9 | |
| R49 Voice and Resonance Disorders | | 182 | 5.1 | |
| R53 Malaise and Fatigue | | 137 | 3.8 | |
| R13 Aphagia and Dysphagia | | 93 | 2.6 | |
| R04 Hemorrhage from Respiratory Passages | | 91 | 2.6 | |
| J31 Chronic Rhinitis, Nasopharyngitis and Pharyngitis | | 89 | 2.5 | |
| J34 Other and Unspecified Disorders of Nose and Nasal Sinuses | | 70 | 2.0 | |
| R10 Abdominal and Pelvic Pain | | 68 | 1.9 | |
| R51 Headache | | 59 | 1.7 | |
| I48 Atrial Fibrillation and Flutter | | 58 | 1.6 | |
| R52 Pain, Not Elsewhere Classified | | 51 | 1.4 | |
| R11 Nausea and Vomiting | | 50 | 1.4 | |
| J38 Diseases of Vocal Cords and Larynx, Not Elsewhere Classified | | 44 | 1.2 | |
| G47 Sleep Disorders | | 43 | 1.2 | |
| J47 Bronchiectasis | | 39 | 1.1 | |
| R55 Syncope and Collapse | | 32 | 0.9 | |
| F45 Somatoform Disorders | | 31 | 0.9 | |
| R00 Abnormalities of Heartbeat | | 30 | 0.8 | |
| J33 Nasal Polyp | | 29 | 0.8 | |
| R12 Heartburn | | 29 | 0.8 | |
| F41 Other Anxiety Disorders | | 28 | 0.8 | |
| R63 Symptoms and Signs Concerning Food and Fluid Intake | | 25 | 0.7 | |
| J39 Other Diseases of Upper Respiratory Tract | | 24 | 0.7 | |
| R09 Other Symptoms and Signs Involving the Circulatory and Respiratory System | | 24 | 0.7 | |
| R42 Dizziness and Giddiness | | 23 | 0.7 | |

One individual might be counted on multiple rows.

**Table E.** Number of individuals with dispense of medicines from ATC groups R01, R03 and A02.

| Individuals with PCC (n=62 963) | Example of use | Medicine retrieved  2016-2018 | Medicine retrieved  2006-2018 |
| --- | --- | --- | --- |
| R01 Drugs for Nasal conditions | Nasal conditions and allergic rhinitis | 12,764 | 22,707 |
| R03 Drugs for Obstructive Pulmonary disease | Asthma + COPD | 22,152 | 23,143 |
| A02 Drugs for acid related disorders | GERD | 11,858 | 17,304 |
| Total | | 26,632 | 31,969 |
|  | |  |  |

One patient may be counted on multiple rows.

**Table F.** Co-diagnosis at R05 visits 2016-2018.

| Co-diagnosis at R05 visit 2016-2018 | | | | |
| --- | --- | --- | --- | --- |
| Diagnosis | **Individuals** | | | **%** |
| R06 Abnormalities of Breathing | | 737 | 20.7 | |
| J30 Vasomotor and Allergic Rhinitis | | 413 | 11.6 | |
| R50 Fever of Other and Unknown Origin | | 374 | 10.5 | |
| J45 Asthma | | 369 | 10.4 | |
| R07 Pain in Throat and Chest | | 285 | 8.0 | |
| K21 Gastro-Esophageal Reflux Disease | | 246 | 6.9 | |
| R49 Voice and Resonance Disorders | | 182 | 5.1 | |
| R53 Malaise and Fatigue | | 137 | 3.8 | |
| R13 Aphagia and Dysphagia | | 93 | 2.6 | |
| R04 Hemorrhage from Respiratory Passages | | 91 | 2.6 | |
| J31 Chronic Rhinitis, Nasopharyngitis and Pharyngitis | | 89 | 2.5 | |
| J34 Other and Unspecified Disorders of Nose and Nasal Sinuses | | 70 | 2.0 | |
| R10 Abdominal and Pelvic Pain | | 68 | 1.9 | |
| R51 Headache | | 59 | 1.7 | |
| I48 Atrial Fibrillation and Flutter | | 58 | 1.6 | |
| R52 Pain, Not Elsewhere Classified | | 51 | 1.4 | |
| R11 Nausea and Vomiting | | 50 | 1.4 | |
| J38 Diseases of Vocal Cords and Larynx, Not Elsewhere Classified | | 44 | 1.2 | |
| G47 Sleep Disorders | | 43 | 1.2 | |
| J47 Bronchiectasis | | 39 | 1.1 | |
| R55 Syncope and Collapse | | 32 | 0.9 | |
| F45 Somatoform Disorders | | 31 | 0.9 | |
| R00 Abnormalities of Heartbeat | | 30 | 0.8 | |
| J33 Nasal Polyp | | 29 | 0.8 | |
| R12 Heartburn | | 29 | 0.8 | |
| F41 Other Anxiety Disorders | | 28 | 0.8 | |
| R63 Symptoms and Signs Concerning Food and Fluid Intake | | 25 | 0.7 | |
| J39 Other Diseases of Upper Respiratory Tract | | 24 | 0.7 | |
| R09 Other Symptoms and Signs Involving the Circulatory and Respiratory System | | 24 | 0.7 | |
| R42 Dizziness and Giddiness | | 23 | 0.7 | |

One individual might be counted on multiple rows.

**Table G.** Clinic type that sets the cough diagnosis and Clinic type of Cough Medicine prescriber.

| **R05 diagnosis recordings** | | | **Cough medicine prescriptions** | | |
| --- | --- | --- | --- | --- | --- |
| **Clinic type** | **%** | **Clinic type** | | **%** | |
| Internal medicine | 29.5 | Primary care | | 82.5 | |
| Ear, nose and throat | 19.9 | Unknown | | 3.5 |  |
| Lung | 15.4 | Internal medicine | | 3.0 |  |
| Emergency | 12.0 | Ear, nose and throat | | 1.5 |  |
| Allergy | 7.7 | Occupational healthcare | | 1.4 |  |
| Infectious disease | 5.2 | Surgical | | 0.9 |  |
| Others | 10.3 | Others | | 7.2 |  |
| Total | 100.0 | Total | | 100.0 |  |

**Table H.** List of medicines dispensed by the individuals in the national cohort 2016-2018.

| **ATC** | **# Individuals** | **% Individuals** | **# Expeditions** |
| --- | --- | --- | --- |
| R05 Medicines against cough and common cold* | 57,171 | 90.8 | 266,528 |
| R03 Anti-obstructive respiratory medicines | 27,301 | 43.4 | 285,116 |
| A02 Medicines for acidity-related symptoms | 23,300 | 37.0 | 209,155 |
| J01 Anti-bacterials for systemic use | 22,504 | 35.7 | 43,880 |
| R06 Antihistaminic drugs for systemic use | 17,014 | 27.0 | 99,259 |
| R01 Medicines for nasal conditions | 16,787 | 26.7 | 64,547 |
| H02 Corticosteroids for systemic use | 14,127 | 22.4 | 55,403 |
| N03 Antiepileptics | 5,512 | 8.6 | 121,892 |
| J05 Antivirals for systemic use | 3,590 | 5.7 | 15,050 |
| J02 Antimycotic agents for systemic use | 2,495 | 4.0 | 5,692 |
| J07 Vaccines | 852 | 1.4 | 1,235 |
| R02 Medicines for throat and pharynx | 68 | 0.1 | 78 |

*R05 is included in the inclusion criteria, outlined in Table S3.

One patient might be counted on multiple rows.

**Table I.** Number of individuals with possible chronic cough stratified by type of cough medicine and number of dispenses per cough medicines 2016-2018.

| Group | ATC | Name | 1  DISP | 2-3  DISP | 4-6  DISP | >6  DISP | Total |
| --- | --- | --- | --- | --- | --- | --- | --- |
| Exp | R05CB01 | Acetylcysteine | 6,440  20.7 % | 12,090  38.9 % | 7,220  23.2 % | 5,301  17.1 % | 31,051  100.0 % |
| Opiates | R05DA04 | Codeine | 314  17.2 % | 484  26.5 % | 325  17.8 % | 705  38.6 % | 1,828  100.0 % |
| CS/ Exp | R05CB02 | Bromhexine | 225  40.8 % | 131  23.7 % | 75  13.6 % | 121  21.9 % | 552  100.0 % |
| CS/ Exp | R05FB02 | Cough Suppressants and expectorants | 162  47.5 % | 122  35.8 % | 44  12.9 % | 13  3.8 % | 341  100.0 % |
| CS | R05DA07 | Noscapine | 220  80.6 % | 48  17.6 % | 4  1.5 % | 1  0.4 % | 273  100.0 % |
| CS | R05DA20 | Combinations | 1,269  49.8 % | 964  37.8 % | 255  10.0 % | 60  2.4 % | 2,548  100.0 % |
| CS | R05FA02 | Opium derivatives and expectorants | 8,868  25.8 % | 19,224  56.0 % | 5,163  15.0 % | 1,074  3.1 % | 34,329  100.0 % |
| Anti-E | R06AA02 | Dimenhydrinate | 7  36.8 % | 9  47.4 % | 3  15.8 % | 0  0.0 % | 19  100.0 % |
| Total | | | 14,841  26.6 % | 31,454  56.3 % | 12,976  23.2 % | 7,206  12.9 % | 55,823  100.0 % |

One patient may have multiple medicines and be counted on multiple rows. Exp: Expectorants, CS: Cough Suppressant, Anti-E: Anti-emetic.
