# Supplementary material for: Characteristics, demographics, and epidemiology of possible chronic cough in Sweden: A nationwide register-based cohort study
Source: PLoS One. 2024 Jul 24;19(7):e0303804. doi: 10.1371/journal.pone.0303804 (PMC11268580; doi:10.1371/journal.pone.0303804)
Supplement: Attachment — Submitted filename: PONE-D-23-39518.pdf [file pone.0303804.s004.pdf]

PLOS ONE

Characteristics, demographics, and epidemiology of possible chronic cough in Sweden: A nationwide register-based cohort study

--Manuscript Draft--

**Manuscript Number:**	PONE-D-23-39518
**Article Type:**	Research Article
**Full Title:**	Characteristics, demographics, and epidemiology of possible chronic cough in Sweden: A nationwide register-based cohort study
**Short Title:**	Characteristics, demographics, and epidemiology of possible chronic cough in Sweden
**Corresponding Author:**	Lotta Walz, Ph.D<br>MSD Sweden<br>Stockholm, SWEDEN
**Keywords:**	Chronic Cough, epidemiology, nationwide cohort, R05, refractory chronic cough, unexplained chronic cough
**Abstract:**	Abstract<br>Aim: To show clinical characteristics, treatments, and comorbidities in chronic cough in a nationwide cohort.<br>Methods: Two cohorts were created. A national cohort with individuals from two population-based databases; the National Patient Register and Swedish Prescribed Drug Register. Secondly, a regional cohort including primary care data. Adults with at least one cough diagnosis (ICD-10 R05) and/or individuals with ≥2 dispensed prescriptions for relevant cough-medication within the inclusion period, 2016–2018, were identified. Individuals on medications which may instigate cough or suggest acute infection or diagnosed with conditions where cough is a cardinal symptom, were excluded. Those remaining were defined as having possible refractory or unexplained chronic cough (RCC/UCC).<br>Results: Altogether 62,963 individuals were identified with possible RCC/UCC, giving a national prevalence of about 1%. Mean age was 56 years and 60% were females. Many (44%) of the individuals with possible RCC/UCC visited cough relevant specialist clinics during the study period, but less than 20% received a cough diagnosis. A majority (63%) had evidence of RCC/UCC in the 10 years prior to inclusion in the study. In the regional cohort, including primary care data, the prevalence of RCC/UCC was doubled (2%). Cough medicines were mainly prescribed by primary care physicians (82%).<br>Conclusion: Most individuals with possible RCC/UCC sought medical care in primary care, had a long history of cough, with various treatments tried, indicating a substantial burden of the condition. Referrals to specialist care was very rare. The results underline the need for a structured multidisciplinary approach and future therapeutic options.
**Order of Authors:**	Lotta Walz, Ph.D
	Kristoffer Illergård, Ph.D
	Johannes Arpegård, Ph.D
	Cristian Dorbesi, Ph.D
	Henrik Johansson, associate professor
	Össur Emilsson, associate professor
**Additional Information:**	
**Question**	**Response**
**Financial Disclosure**<br><br>Enter a financial disclosure statement that describes the sources of funding for the	Yes

work included in this submission. Review the submission guidelines for detailed requirements. View published research articles from *PLOS ONE* for specific examples.<br><br>This statement is required for submission and **will appear in the published article** if the submission is accepted. Please make sure it is accurate.<br><br>**Funded studies**<br>Enter a statement with the following details:<br>• Initials of the authors who received each award<br>• Grant numbers awarded to each author<br>• The full name of each funder<br>• URL of each funder website<br>• Did the sponsors or funders play any role in the study design, data collection and analysis, decision to publish, or preparation of the manuscript?<br><br>Did you receive funding for this work?	
Please add funding details.<br>as follow-up to "**Financial Disclosure**<br><br>Enter a financial disclosure statement that describes the sources of funding for the work included in this submission. Review the submission guidelines for detailed requirements. View published research articles from *PLOS ONE* for specific examples.<br><br>This statement is required for submission and **will appear in the published article** if the submission is accepted. Please make sure it is accurate.	This study was supported by MSD (Sweden) AB. The funding was used by REVEAL for data extractions, and time spent for aggregation and analysis of data (grant/award number: not applicable).

Powered by Editorial Manager® and ProduXion Manager® from Aries Systems Corporation

**Funded studies**<br>Enter a statement with the following details:<br>• Initials of the authors who received each award<br>• Grant numbers awarded to each author<br>• The full name of each funder<br>• URL of each funder website<br>• Did the sponsors or funders play any role in the study design, data collection and analysis, decision to publish, or preparation of the manuscript?<br><br>Did you receive funding for this work?"	
Please select the country of your main research funder (please select carefully as in some cases this is used in fee calculation).<br>as follow-up to "**Financial Disclosure**<br><br>Enter a financial disclosure statement that describes the sources of funding for the work included in this submission. Review the submission guidelines for detailed requirements. View published research articles from *PLOS ONE* for specific examples.<br><br>This statement is required for submission and **will appear in the published article** if the submission is accepted. Please make sure it is accurate.<br><br>**Funded studies**<br>Enter a statement with the following details:<br>• Initials of the authors who received each award<br>• Grant numbers awarded to each author<br>• The full name of each funder<br>• URL of each funder website<br>• Did the sponsors or funders play any role in the study design, data collection and analysis, decision to publish, or preparation of the manuscript?<br><br>Did you receive funding for this work?"	SWEDEN - SE
**Competing Interests**	Conflicts of interest:The authors report no conflicts of interest in this work, except that

Use the instructions below to enter a competing interest statement for this submission. On behalf of all authors, disclose any competing interests that could be perceived to bias this work—acknowledging all financial support and any other relevant financial or non-financial competing interests.<br><br>This statement is required for submission and **will appear in the published article** if the submission is accepted. Please make sure it is accurate and that any funding sources listed in your Funding Information later in the submission form are also declared in your Financial Disclosure statement.<br><br>View published research articles from *PLOS ONE* for specific examples.<br><br>**NO authors have competing interests**<br><br>Enter: *The authors have declared that no competing interests exist.*<br><br>**Authors with competing interests**<br><br>Enter competing interest details beginning with this statement:<br><br>*I have read the journal's policy and the authors of this manuscript have the following competing interests: [insert competing interests here]*<br><br>* typeset	LW is employed by MSD (Sweden) AB, but with no stock ownership. ÖE has received honoraria from MSD and AstraZeneca for advisory work, unrelated to this manuscript.
**Ethics Statement**<br><br>Enter an ethics statement for this submission. This statement is required if the study involved:<br><br>• Human participants<br>• Human specimens or tissue<br>• Vertebrate animals or cephalopods<br>• Vertebrate embryos or tissues	Patient and Public Involvement<br>This study is in part inspired by the recent guidelines on chronic cough, where patients were involved, highlighting the need for better epidemiological studies. Patients were not involved in the design of this observational register study. Unique Personal Identity Numbers were used for linking individual data between the sources. Thereafter, the data were pseudonymized by replacing the Personal Identity Numbers with a serial number. This process was performed at the NBHW before the research group received access to data. As the data was pseudonymized healthcare register data only, consent was not needed from the individuals, as confirmed by the ethics committee. The study was approved by the Swedish authority for Ethical approval Review Board (Ref: 2019-

• Field research<br><br>Write "N/A" if the submission does not require an ethics statement.<br><br>General guidance is provided below. Consult the submission guidelines for detailed instructions. **Make sure that all information entered here is included in the Methods section of the manuscript.**	06060)<br>Approval attached.

Powered by Editorial Manager® and ProduXion Manager® from Aries Systems Corporation

**Format for specific study types**<br><br>**Human Subject Research (involving human participants and/or tissue)**<br>• Give the name of the institutional review board or ethics committee that approved the study<br>• Include the approval number and/or a statement indicating approval of this research<br>• Indicate the form of consent obtained (written/oral) or the reason that consent was not obtained (e.g. the data were analyzed anonymously)<br><br>**Animal Research (involving vertebrate animals, embryos or tissues)**<br>• Provide the name of the Institutional Animal Care and Use Committee (IACUC) or other relevant ethics board that reviewed the study protocol, and indicate whether they approved this research or granted a formal waiver of ethical approval<br>• Include an approval number if one was obtained<br>• If the study involved *non-human primates*, add *additional details* about animal welfare and steps taken to ameliorate suffering<br>• If anesthesia, euthanasia, or any kind of animal sacrifice is part of the study, include briefly which substances and/or methods were applied<br><br>**Field Research**<br><br>Include the following details if this study involves the collection of plant, animal, or other materials from a natural setting:<br>• Field permit number<br>• Name of the institution or relevant body that granted permission	
**Data Availability**<br><br>Authors are required to make all data underlying the findings described fully available, without restriction, and from the time of publication. PLOS allows rare exceptions to address legal and ethical concerns. See the PLOS Data Policy and FAQ for detailed information.	No - some restrictions will apply

A Data Availability Statement describing where the data can be found is required at submission. Your answers to this question constitute the Data Availability Statement and **will be published in the article**, if accepted.<br><br>**Important:** Stating 'data available on request from the author' is not sufficient. If your data are only available upon request, select 'No' for the first question and explain your exceptional situation in the text box.<br><br>Do the authors confirm that all data underlying the findings described in their manuscript are fully available without restriction?	
**Describe where the data may be found in full sentences. If you are copying our sample text, replace any instances of XXX with the appropriate details.**<br><br>• If the data are **held or will be held in a public repository**, include URLs, accession numbers or DOIs. If this information will only be available after acceptance, indicate this by ticking the box below. For example: *All XXX files are available from the XXX database (accession number(s) XXX, XXX.*).<br>• If the data are all contained **within the manuscript and/or Supporting Information files**, enter the following: *All relevant data are within the manuscript and its Supporting Information files.*<br>• If neither of these applies but you are able to provide **details of access elsewhere**, with or without limitations, please do so. For example:<br><br>*Data cannot be shared publicly because of [XXX]. Data are available from the XXX Institutional Data Access / Ethics Committee (contact via XXX) for researchers who meet the criteria for access to confidential data.*<br><br>*The data underlying the results presented in the study are available from (include the name of the third party*	Data cannot be shared publicly because it cannot be fully anonymised. Data are available from the authors upon a reasonable request, and in accordance with ethics applications, for researchers who meet the criteria for access to confidential data.

and contact information or URL).<br>• This text is appropriate if the data are owned by a third party and authors do not have permission to share the data.<br><br>* typeset	
Additional data availability information:	

Manuscript
Click here to access/download;Manuscript;Manuscript cough registry study - Plos One.docx

Characteristics, demographics, and epidemiology of possible chronic cough in Sweden: A nationwide register-based cohort study

Corresponding author:

Lotta Walz

MSD (Sweden) AB, Gävlegatan 22 SE 113 30 Stockholm, Sweden

Mobil phone + 46 70 636 33 23

Lotta.walz@merck.com

Lotta Walz,[1] Kristoffer Illergård,[2] Johannes Arpegård,[2] Cristian Dorbesi[2] Henrik Johansson,[3,4] Össur Ingi Emilsson[5,6]

1. MSD (Sweden) AB, Gävlegatan 22 SE 113 30 Stockholm, Sweden
2. Division of research, informatics & visualization, Reveal AB, Stockholm, Sweden.
3. Department of Medical Sciences, Clinical Physiology, Uppsala University, Uppsala, Sweden
4. Department of Women's and Children's Health, Physiotherapy, Uppsala University, Uppsala, Sweden.
5. Department of Medical Sciences, Respiratory, allergy and sleep research, Uppsala University, Uppsala, Sweden
6. Respiratory medicine and allergology, Akademiska sjukhuset, Uppsala, Sweden

The study was approved by the Swedish authority for Ethical approval Review Board (Ref: 2019-06060).

Keywords: Chronic Cough, epidemiology, nationwide cohort, R05, refractory chronic cough, unexplained chronic cough

Acknowledgement

We want to thank Prof Eva Millqvist for valuable and important input to the study protocol. Appreciations to MSD (Sweden) AB for the unrestricted funding to support time spent for aggregation and analysis of data.

Contribution

LW and ÖE headed the study design, with contributions from all co-authors. K.I., J.A. and C.D. had full access to all data in the study and takes responsibility for the integrity of the data and the accuracy of the data analysis. Every analysis and all tables are performed individually by several of the authors and reviewed by all authors. LW, ÖE and HJ headed the manuscript writing. All authors revised and contributed to the manuscript and approved the final form of the manuscript for submission.

Take-home message

Individuals with possible chronic cough identified in Swedish registers have a long history of cough, with many treatments tried, indicating a marked burden of the condition. This shows a need for a better and more structured approach to chronic cough.

Abstract

Aim: To show clinical characteristics, treatments, and comorbidities in chronic cough in a nationwide cohort.

Methods: Two cohorts were created. A national cohort with individuals from two population-based databases; the National Patient Register and Swedish Prescribed Drug Register. Secondly, a regional cohort including primary care data. Adults with at least one cough diagnosis (ICD-10 R05) and/or individuals with ≥2 dispensed prescriptions for relevant

cough-medication within the inclusion period, 2016–2018, were identified. Individuals on medications which may instigate cough or suggest acute infection or diagnosed with conditions where cough is a cardinal symptom, were excluded. Those remaining were defined as having possible refractory or unexplained chronic cough (RCC/UCC).

Results: Altogether 62,963 individuals were identified with possible RCC/UCC, giving a national prevalence of about 1%. Mean age was 56 years and 60% were females. Many (44%) of the individuals with possible RCC/UCC visited cough relevant specialist clinics during the study period, but less than 20% received a cough diagnosis. A majority (63%) had evidence of RCC/UCC in the 10 years prior to inclusion in the study. In the regional cohort, including primary care data, the prevalence of RCC/UCC was doubled (2%). Cough medicines were mainly prescribed by primary care physicians (82%).

Conclusion: Most individuals with possible RCC/UCC sought medical care in primary care, had a long history of cough, with various treatments tried, indicating a substantial burden of the condition. Referrals to specialist care was very rare. The results underline the need for a structured multidisciplinary approach and future therapeutic options.

Introduction

Chronic cough, defined as cough lasting >8 weeks, is common in the general population, but difficult to treat.[1] Between 10-38% of individuals in a respiratory outpatient clinic in the USA have chronic cough,[1] and in Swedish general population the prevalence of habitual non-productive cough is estimated to be 7-11%.[2,3] Severe chronic cough causes a major decline in health-related quality of life, with comorbidities including incontinence, cough syncope and dysphonia, leading to social isolation and depression.[4] Also, chronic cough is associated with more sick leave and lower work ability.[2] In spite of this, individuals with chronic cough often report their condition being inadequately appreciated in healthcare.[4,5]

Individuals with chronic cough also seem to seek healthcare to a varying degree.[6] Since there are no specific diagnostic or treatment protocols for chronic cough, and specialist cough clinics are rare, there is a risk for potential discrepancies in the treatments provided for chronic cough.[7] Around half of individuals with chronic cough may still have chronic cough five years after initial evaluation.[8]

The terms unexplained or refractory chronic cough (UCC/RCC) are widely used to describe chronic cough that either has no identifiable cause (such as asthma, gastroesophageal reflux disease (GERD), or rhinitis), or doesn't get better despite well-conducted treatment, respectively.[2,3] Currently, only a few medical treatments recommended by guidelines for cough, some being potentially addictive medications such as morphine.[9] A recent register-based study from Denmark used prescription and diagnosis healthcare register data to study chronic cough among patients seeking healthcare.[10] In that study, only 3% of the adult population could be identified as having possible chronic cough (PCC), significantly lower than reported in the general population.[10] This could be caused by a lack of clinical acknowledgement of cough, or patients not seeking healthcare. A few shortcomings in the

study were that diagnosis data was not available from primary care, lacked analysis of differences between care providers or counties, and the duration of cough.[10] Therefore, better knowledge on the current real-world diagnosis and treatment of individuals with chronic cough is needed, to increase the awareness and improve healthcare for individuals with chronic cough.

The aim was to describe the real-world epidemiology of chronic cough in Sweden by using national register data. More specifically, to describe clinical characteristics and demographics as well as healthcare utilization including medical treatment of a population with possible chronic cough.

Methods

This is a descriptive nationwide observational register-based cohort study, conducted on an adult population identified with possible chronic cough in Sweden from January 2016 – December 2018.

Two different cohorts were created from data available in population-based registers (National Patient Register and Swedish Prescribed Drug Register) as previously described in a Danish cohort,[10] with slight differences as described below (Figure 1).

The national cohort included individuals from two Swedish population-based databases: 1) The National Patient Register, covering information on every hospitalization or non-primary care outpatient visits of all Swedish citizens and residents with a 100% coverage. For the included patients information on diagnoses, using ICD-10 (International classification of disease), were identified;[11] 2) The Swedish Prescribed Drug Register, containing information on all prescribed medicines and pharmaceutical aids dispensed at Swedish pharmacies since June 2005 (100% coverage).[12]

The regional cohort was created by selecting individuals residing in Östergötland county, Sweden, from the national cohort then adding individuals identified with PCC from the county's primary care data. The same inclusion and exclusion criteria were applied.

Data covering earlier R05 diagnoses, comorbidities, and dispensed prescriptions of relevant chough medicine from 2006-2015 for the individuals in the study identified were retrospectively assessed from the same registers.

Unique personal identity numbers (PIN) were used for linking individual data between the sources. Thereafter, the data were pseudonymized by replacing the PIN with a serial number. This process was performed at the National Board of Health and Welfare before the research group received access to data. As the data was pseudonymized, healthcare register data only, consent was not needed from the individuals, as confirmed by the ethics committee. The study was approved by the Swedish authority for Ethical approval Review Board (Ref: 2019-06060)

Figure 1. Flowchart illustrating the process of identifying individuals with possible chronic cough from Swedish registers. NPR: National Patient Register; SPDR: Swedish Prescribed Drug Register; PC: Primary Care; ÖG: Östergötland; TBC: Tuberculosis; ACEi: Angiotensin Converting Enzyme inhibitor.

Inclusion and exclusion criteria

Inclusion criteria were: Individuals ≥18 of age at date of inclusion, with either minimum one ICD-10 diagnosis code of R05 (cough/cough with no further specification) recorded at a healthcare visit or ≥2 dispenses of relevant cough-medication (as listed in Table S1) at least 8 weeks apart within the inclusion period, 1st January 2016 – 31st dec 2018. This definition was used to capture chronic cough (lasting more than 8 weeks).

Individuals were excluded if they met any of the following criteria: 1) Medications which can instigate cough or suggest acute infection, i.e. ACEi or antibiotics, respectively; 2) Conditions where cough is a cardinal symptom, such as: acute cough, pneumonia, tuberculosis, chronic

obstructive pulmonary disease (COPD), lung cancer and interstitial lung disease, identified by ICD-10 codes. For acute conditions such as pneumonia or acute upper airway infection and antibiotic medications, the event date needed to be within 8 weeks (before or after) from the cough diagnosis or dispensed cough medicine for the subject to be excluded. For other conditions not considered acute, such as cancer, tuberculosis, COPD, other lung diseases or treatment with ACEi, the event could take place at any time during the inclusion period 2016-2018. The exclusion steps and the exclusion groups are summarized in supplementary data (Tables S2 and S3).

Possible refractory /unexplained chronic cough

All eligible individuals in the final cohort were stratified into either possible refractory chronic cough (RCC) or possible unexplained chronic cough (UCC). Individuals were defined as having possible RCC if diagnosed at least once with any of the cough associated co-diagnoses (asthma, GERD, rhinitis) and/or treated with any of the drugs associated with these conditions (at least two dispenses) during the inclusion period. Otherwise, individuals were classified as having possible UCC. The number of individuals per diagnosis are provided in Supplementary table S4 and the number of individuals per associated drugs are provided in Supplementary table S5.

Cough-relevant specialist clinics were identified as each clinical type that was responsible for more than 5% of the total number of R05 diagnoses in the national data set. In this cohort cough relevant specialist clinics were specialties of Internal medicine, Ear, nose and throat, Lung, Emergency, Infectious disease, or Allergy.

Mean age and standard deviation at the date of inclusion was calculated, assuming a normalized age distribution. General population data from Statistics Sweden was used to calculate prevalence.

Results

National cohort

During the study period 256,046 individuals were initially identified with at least one ICD-10 diagnosis code of R05 and / or ≥2 dispenses of relevant cough-medication in the national cohort.

After applying exclusion criteria (Figure 1, Table S2), a total of 62,963 individuals were identified with PCC (corresponding to prevalence of 787/100,000 among inhabitants of age ≥18 years), of which 80% were identified from cough medicine dispenses only (Figure 2). The mean age was 56 years, the majority were female, and 56% had possible UCC (Table 1). The individuals identified from cough medicine dispenses only were on average 9 years older than those identified with cough diagnosis only (Table 1). Age was normally distributed, with few individuals in the oldest and youngest age groups (Figure 3 and Supplementary Figure S1). The frequency of cough medicine dispenses varied, with 37% of the individuals having four dispenses or more during the study period (Figure 4).

Figure 2. Two Venn diagrams illustrating the number of individuals with possible chronic cough identified by cough medicine dispenses and/or cough diagnosis. In turquoise individuals identified with ≥2 dispenses of relevant cough-medication in the Swedish Prescribed Drug Register, in yellow individuals with cough diagnosis (R05) a) the national cohort with cough diagnosis (R05) from the Swedish National Patient Register and b) the regional cohort with cough diagnosis (R05) from the National Patient Register and the primary care register.

Table 1. Characteristics of individuals with possible chronic cough.

	**National cohort**				**Regional cohort**
**Parameters**	Cough medicine only	Cough Diagnosis only	Cough Medicine & Cough Diagnosis	Total	Total
Individuals (n)	50,474	10,794	1,695	62,963	6,402

Individuals (% of cohort)	80.2 %	17.1 %	2.7 %	100 %	100 %
Woman (%)	60.8 %	56.6 %	61.4 %	60.1 %	58.2 %
Age (mean (SD))	58 (18.0)	49 (17.9)	55 (16.4)	56 (18.2)	51 (18.0)
Age >65 (%)	38.2 %	21.3 %	30.3 %	35.1 %	24.4 %
RCC (%)	44.1 %	41.5 %	62.2 %	44.2 %	33.3 %
Has Asthma* (%)	10.2 %	12.4 %	16.9 %	10.8 %	13.1 %
Has Rhinitis* (%)	7.1 %	12.4 %	14.6 %	8.1 %	9.0 %
Has GERD* (%)	5.1 %	7.9 %	14.4 %	5.8 %	8.5 %
UCC (%)	55.9 %	58.5 %	37.8 %	55.8 %	66.7 %

*Recorded diagnosis during 2016-2018

GERD: Gastroesophageal reflux disease; RCC: Refractory chronic cough; UCC: Unexplained chronic cough.

Figure 3. Distribution of age and gender for individuals with possible chronic cough in the national cohort.

Figure 4. a) Proportion of individuals with possible chronic cough stratified by number of visits with a R05 diagnosis. b) Proportion of individuals

Some variation was seen between counties regarding the prevalence of cough medicine dispenses and cough diagnosis. Individuals with cough medicine ranged from 499/100,000 inhabitants to 819/100,000 inhabitants, and individuals with cough diagnosis ranged from 99/100,000 inhabitants to 189/100,000 inhabitants (Supplementary figure S2).

Even though 44% of individuals with possible chronic cough had visited any cough relevant specialist clinic during the study period, only 41% of these individuals received a cough diagnosis code, meaning that less than 20% of the whole PCC cohort received a cough diagnosis (ICD-10 code R05, n=12 489) (Figure 5). Of the individuals who were diagnosed with cough, the majority did not receive any other diagnosis on the same visit (72%). The most common co-diagnosis was R06 (abnormalities of breathing, 21%). Other co-diagnoses are reported in Supplementary table S6.

Figure 5. a) Individuals visiting cough relevant specialist clinics, stratified by number of visits per patient 2016-2018. b) Number of cough diagnosis recordings at cough relevant specialist clinics cough relevant clinics.

Cough medicine were mainly prescribed in primary care (82.5%) (Supplementary table S7). Treatment with other medications was rather common, where dispensed prescriptions for obstructive respiratory disease were most common (27,301 of those with PCC (43%)). Also, 23,300 (37%) had dispensed prescriptions of anti-acid medicine, 22,504 (36%) had dispensed prescriptions of antibiotics more than 8 weeks prior to or after the date of inclusion, 17,014 (27%) had dispensed prescriptions of systemic antihistamines, and 16,787 (27%) had dispensed prescriptions of nasal medicine. A substantial number of individuals, 5,512 (9 %), had a dispensed prescription of anti-epileptic medicine (N03) but without epilepsy diagnosis. Further details on dispensed prescriptions are reported in Supplementary table S8.

When looking at opioids specifically, 36,838 (59%) individuals had dispensed any of the opioids (ATC code R05DA04, R05DA20 or R05FA02), and 7,653 (20%) individuals had four or more expeditions in the three-year study period (Supplementary table S9).

The national study cohort was then retrospectively analysed, where a majority (63%) of individuals with PCC in 2016-2018 had evidence of a history of cough in the 10 years-period prior to the inclusion, and most commonly already at 10 years prior to inclusion (Figure 6a). More than 30% of the individuals had used cough medicine at least 4 of the prior 10 years, and 45% had dispensed at least 2 prescriptions of cough medicine per year (Figure 6b).

Figure 6. History of chronic cough a) First identification of possible chronic cough in the 10 years-period prior inclusion in the national cohort. b) Number of years ‘active’ and diagnoses per active year. A patient is considered to have an ‘active’ cough year if they have drug dispenses or cough diagnosis recordings.

Regional cohort

In the regional cohort, where data on diagnoses from primary care was included, the prevalence of PCC was 1.8% (or 1,760 /100,000 inhabitants), mostly because of more frequent cough diagnosis (Figure 2b). The age and gender distribution were similar to the national cohort (Table 1). However, the prevalence in different age groups differed between the regional and national cohort. In the national cohort, the prevalence increased with increasing age groups (18-39, 40-69, ≥70 years), while in the regional cohort, the highest prevalence was seen among those 40-69 years of age (Table 2). A small proportion of individuals identified with PCC in primary care (3.5%) had a referral including a cough diagnosis to a specialist in secondary care.

Table 2. Number of individuals in possible chronic cough cohorts and background cohorts and prevalence for different age groups.

Cohort	Age	PCC	Population	Prevalence
National	All ages	62,963	80.03 K	787
	18-39	12,533	28.53 K	440
	40-69	34,190	36.94 K	926
	>=70	16,227	14.58 K	1 113
Regional	All ages	6,402	3.63 K	1762
	18-39	1,933	1.31 K	1 477
	40-69	3,384	1.65 K	2 056
	>=70	1,085	0.68 K	1 598

PCC: Possible chronic cough.

Discussion

In this observational study, possible chronic cough (PCC) was identified in 0.8% of the Swedish adult population, using national pharmacy and secondary care registers. When primary care register data from a single healthcare region was added to the national cohort, the prevalence of PCC in that region increased to 1.8%. The highest prevalence was found in middle-aged adults; higher among females than males. Individuals with PCC were mostly managed in primary care, where also most cough medications were prescribed. Often, the cough medication included opioids. One fourth of the PCC population had two or more visits to cough-relevant clinics during the inclusion period of three years, but only a minority received a cough diagnosis. Altogether, 44% of those with PCC had an identifiable cause of chronic cough (refractory chronic cough, RCC). Most of the individuals (63%) with PCC had register-based evidence of chronic cough in the 10 year-period prior to inclusion. This supports previous findings that RCC/UCC most likely persist for years, causing substantial morbidity and impaired health status.[2,5] A European survey on 1120 individuals with chronic cough determined that the median duration of cough was 2 to 5 years, 72% of respondents had visited their doctor more than 3 times, but only 53% had received a diagnosis.[4]

We found that more than 80% of those with dispensed prescriptions of cough medication received their prescription from primary care. In line with recently published primary care data from the UK, our results support that individuals with chronic cough usually seek medical attention from a healthcare provider in primary care as the first and often only instance.[13,14]

The prevalence of PCC in the present study (0.8%) was lower than in many questionnaire-based general population studies. For example, a recent epidemiological review

found a prevalence of 9.6% in the general population.[15] On the other hand, studies that have used electronic healthcare records or register data have found a lower prevalence, more similar to our findings. A study using electronic healthcare records register in California, USA, found a chronic cough prevalence of 1.0%.[16] A similar study from the UK using both primary and secondary care data found a prevalence of 2.0%, without excluding individuals with COPD.[13] Possible explanations for these discrepancies in prevalence numbers may be that individuals with any of a variety of possible causes of chronic cough were excluded in the current analysis (such as COPD, treatment with ACE inhibitors, treatment with antibiotics, cancer, tuberculosis, and pneumonia), which may have led to an underestimated PCC population.[17] Notable is that in our cohort, more than 42% of the individuals with at least one ICD-10 diagnosis code of R05 or ≥2 dispenses of relevant cough-medication were treated with ACE inhibitors.

Additionally, there is no adequate and commonly agreed method to define chronic cough in epidemiological studies.[18] A recent analysis by Hull *et al.* argued that only using structured data to identify individuals with chronic cough leads to an underestimation of the actual number since there are no approved treatments or an approved ICD-10 code for RCC/UCC (at least at that time).[13] Also, cough is for many clinicians regarded more as a symptom than a distinct medical condition or disease, and therefore not diagnosed and coded as such.[14] This is also supported by the present study, where individuals with possible chronic cough rarely seemed to receive a cough diagnosis on visits to cough relevant clinics.

Another possible explanation for the lower prevalence in register-based data is that individuals with chronic cough have stopped seeking healthcare for their cough, likely because of a lack of treatment modalities.[9] Therefore, these individuals cannot be identified from register data as the time period analysed is relatively short. Indeed, our retrospective

data, covering a 10 years-period prior to the inclusion period, showed that the majority of individuals had evidence of PCC 10 years before the inclusion date.

The higher prevalence of PCC in females and in elderly individuals is consistent with findings in other studies.[4,18] The most common comorbidities in the cohort of possible refractory chronic cough were gastroesophageal reflux disease (GERD), allergic rhinitis and asthma, also consistent with the current knowledge.[9]

A considerable number of individuals, 36,838, of the adult population in Sweden were identified with dispensed prescriptions of opioids intended for chronic cough (i.e. around 0.5%). The prescription of opioids is of increasing concern because of addiction-related problems.[19] Other non-addictive treatments need to be sought for this evidently large group of patients.

Most of the individuals in this study had multiple cough medicine dispenses at least eight weeks apart, and almost half of the population had four or more dispenses during the study period, indicating significant chronic cough. In addition, 8.6% of the individuals had dispensed more than 2 prescriptions of cough-relevant anti-epileptics but with no records of associated diagnosis. Only 2% of individuals with at least four dispenses of cough medicines, had a recorded cough diagnosis (R05) in this study. This may in part reflect the lack of an appropriate ICD-10 code at the time. In line with this, a survey performed by the European Lung Foundation found that most individuals (>70%) with long lasting cough had visited their specialist physician 3 times or more. Of these, only 53% had received a cough diagnosis.[4] A recently established ICD-10 code specifically for chronic cough will hopefully lead to more individuals receiving an appropriate diagnosis.[9]

Strengths and Limitations

The nationwide cohort included a large sample of a well-defined group of patients, which is the main strength of this study. Both the prescription and patient registers are validated on a national level, giving the collected real-world data high level of credibility and almost 100% coverage.[11,12] To our knowledge, this is one of very few such studies describing a nationwide population with possible refractory or unexplained chronic cough.

However, there were also some methodological challenges. As inherent to register studies, recorded data was limited by the quality of routinely entered data. As cough is often seen as a symptom rather than a disease, the use of the ICD-10 code R05 for cough is likely underused, especially in RCC. Furthermore, over-the-counter sales of cough medicines is not recorded in the national registers and thus chronic cough individuals who only self-medicate will not be captured.

The lack of data from primary care for many Swedish healthcare regions led to a significant number of missing individuals with PCC, as seen by the doubled prevalence in a region with primary care data. However, the characteristics of the patient groups identified with and without primary care data were in most aspects similar.

Identifying RCC/UCC patients based on register data only has some challenges.[20] Some of the information such as adequate treatment of underlying conditions (for RCC) or diagnostic workup process (for UCC) is not available from the register data. Repeated acute cough episodes may have been misclassified as chronic cough, or vice versa. There is a need for improved diagnostic codes characterizing chronic cough to minimize misclassification. Smoking and obesity is also known to be associate with chronic cough.[18,20,21] Unfortunately, Swedish registry data does not include BMI or identify smokers, and therefore we could not explore the impact of obesity and smoking on chronic cough.

Nevertheless, the data provide an important presentation of the characteristics of a nationwide population with possible refractory or unexplained chronic cough, and how chronic cough has been managed in Sweden without formal diagnostic or treatment guidelines.

Conclusions

This nationwide observational register-based study contributes to a better understanding of individuals affected by refractory or unexplained chronic cough. Possible chronic cough (PCC) is more prevalent in females and increases with age with a peak around 40-69 years. The majority of individuals with PCC had frequently dispensed medical prescriptions, and a long history of PCC although often without receiving a formal cough diagnosis. Pharmacological therapy often included the use of opioids. Most individuals with PCC were treated in primary care, without secondary care specialist referrals. This signifies a need of increased awareness of the condition, better treatments, and improved access to specialist care. Further studies are needed to establish how well register-based cohorts of PCC represent chronic cough individuals in general, and how severely they are affected by their cough, to further increase the understanding of this debilitating condition and improve patient care.

Acknowledgement

We want to thank Prof Eva Millqvist for valuable and important input to the study protocol. Appreciations to MSD (Sweden) AB for the unrestricted funding to support time spent for aggregation and analysis of data.

References

1. Chung KF, Pavord ID. Prevalence, pathogenesis, and causes of chronic cough. Lancet. 2008;371:1364–74.

2. Johansson H, Johannessen A, Holm M, et al. Prevalence, progression and impact of chronic cough on employment in Northern Europe. Eur Respir J. 2021;57:2003344.

3. Lúdvíksdóttir D, Björnsson E, Janson C, et al. Habitual coughing and its associations with asthma, anxiety, and gastroesophageal reflux. Chest. 1996;109:1262–8.

4. Chamberlain SA, Garrod R, Douiri A, et al. The impact of chronic cough: a cross-sectional European survey. Lung. 20150319th ed. 2015;193:401–8.

5. Morice AH, Millqvist E, Belvisi MG, et al. Expert opinion on the cough hypersensitivity syndrome in respiratory medicine. Eur Respir J. 20140819th ed. 2014;44:1132–48.

6. Koskela HO, Lätti AM, Pekkanen J. Risk factors for repetitive doctor's consultations due to cough: a cross-sectional study in a Finnish employed population. BMJ Open. 2019;9:e030945.

7. Puente-Maestu L, Molina-París J, Trigueros JA, et al. A Survey of Physicians' Perception of the Use and Effectiveness of Diagnostic and Therapeutic Procedures in Chronic Cough Patients. Lung. 2021;199:507–15.

8. Koskela HO, Lätti AM, Purokivi MK. Long-term prognosis of chronic cough: a prospective, observational cohort study. BMC Pulm Med. 20171121st ed. 2017;17:146.

9. Morice AH, Millqvist E, Bieksiene K, et al. ERS guidelines on the diagnosis and treatment of chronic cough in adults and children. Eur Respir J. 2020;55:1901136.

10. Backer V, Porsborg A, Hansen V, et al. A register-based study: cough - a frequent phenomenon in the adult population. BMC Pulm Med. 2022;22:426.

11. Ludvigsson JF, Andersson E, Ekbom A, et al. External review and validation of the Swedish national inpatient register. BMC Public Health. 20110609th ed. 2011;11:450.

12. Wettermark B, Hammar N, Fored CM, et al. The new Swedish Prescribed Drug Register--opportunities for pharmacoepidemiological research and experience from the first six months. Pharmacoepidemiol Drug Saf. 2007;16:726–35.

13. Hull JH, Langerman H, Ul-Haq Z, et al. Burden and impact of chronic cough in UK primary care: a dataset analysis. BMJ Open. 2021;11:e054832.

14. Weiner M, Dexter PR, Heithoff K, et al. Identifying and Characterizing a Chronic Cough Cohort Through Electronic Health Records. Chest. 2021;159:2346–55.

15. Song W-J, Chang Y-S, Faruqi S, et al. The global epidemiology of chronic cough in adults: a systematic review and meta-analysis. Eur Respir J. 2015;45:1479–81.

16. Zeiger RS, Xie F, Schatz M, et al. Prevalence and Characteristics of Chronic Cough in Adults Identified by Administrative Data. TPJ. 2020;24:20.022.

17. Landt E, Çolak Y, Lange P, et al. Chronic Cough in Individuals With COPD. Chest. 2020;157:1446–54.

18. Çolak Y, Nordestgaard BG, Laursen LC, et al. Risk Factors for Chronic Cough Among 14,669 Individuals From the General Population. Chest. 2017;152:563–73.

19. Martins SS, Keyes KM, Storr CL, et al. Birth-cohort trends in lifetime and past-year prescription opioid-use disorder resulting from nonmedical use: results from two national surveys. J Stud Alcohol Drugs. 2010;71:480–7.

20. Holden SE, Morice A, Birring SS, et al. Cough presentation in primary care and the identification of chronic cough: a need for diagnostic clarity? Current Medical Research and Opinion. 2020;36:139–50.

21. Landt EM, Çolak Y, Nordestgaard BG, et al. Risk and impact of chronic cough in obese individuals from the general population. Thorax. 2022;77:223–30.

Figure legends

Figure 1. Flowchart illustrating the process of identifying individuals with possible chronic cough from Swedish registers. NPR: National Patient Register; SPDR: Swedish Prescribed Drug Register; PC: Primary Care; ÖG: Östergötland; TBC: Tuberculosis; ACEi: Angiotensin Converting Enzyme inhibitor.

Figure 2. Two Venn diagrams illustrating the number of individuals with possible chronic cough identified by cough medicine dispenses and/or cough diagnosis. In turquoise individuals identified with ≥2 dispenses of relevant cough-medication in the Swedish Prescribed Drug Register, in yellow individuals with cough diagnosis (R05) a) the national cohort with cough diagnosis (R05) from the Swedish National Patient Register and b) the regional cohort with cough diagnosis (R05) from the National Patient Register and the primary care register.

Figure 3. Distribution of age and gender for individuals with possible chronic cough in the national cohort.

Figure 4. a) Proportion of individuals with possible chronic cough stratified by number of visits with a R05 diagnosis. b) Proportion of individuals with possible chronic cough stratified by number of cough medicines dispensations 2016-2018.

Figure 5. a) Individuals visiting cough relevant specialist clinics, stratified by number of visits per patient 2016-2018. b) Number of cough diagnosis recordings at cough relevant specialist clinics cough relevant clinics.

Figure 6. History of chronic cough a) First identification of possible chronic cough in the 10 years-period prior inclusion in the national cohort. b) Number of years 'active' and diagnoses per active year. A patient is considered to have an 'active' cough year if they have drug dispenses or cough diagnosis recordings.

Tables

Table 1. Characteristics of individuals with possible chronic cough.

	National cohort				Regional cohort
**Parameters**	Cough medicine only	Cough Diagnosis only	Cough Medicine & Cough Diagnosis	Total	Total
Individuals (n)	50,474	10,794	1,695	62,963	6,402
Individuals (% of cohort)	80.2 %	17.1 %	2.7 %	100 %	100 %
Woman (%)	60.8 %	56.6 %	61.4 %	60.1 %	58.2 %
Age (mean (SD))	58 (18.0)	49 (17.9)	55 (16.4)	56 (18.2)	51 (18.0)
Age >65 (%)	38.2 %	21.3 %	30.3 %	35.1 %	24.4 %
RCC (%)	44.1 %	41.5 %	62.2 %	44.2 %	33.3 %
Has Asthma* (%)	10.2 %	12.4 %	16.9 %	10.8 %	13.1 %
Has Rhinitis* (%)	7.1 %	12.4 %	14.6 %	8.1 %	9.0 %
Has GERD* (%)	5.1 %	7.9 %	14.4 %	5.8 %	8.5 %
UCC (%)	55.9 %	58.5 %	37.8 %	55.8 %	66.7 %

*Recorded diagnosis during 2016-2018

GERD: Gastroesophageal reflux disease; RCC: Refractory chronic cough; UCC: Unexplained chronic cough.

Table 2. Number of individuals in possible chronic cough cohorts and background cohorts and prevalence for different age groups.

Cohort	Age	PCC	Population	Prevalence
National	All ages	62,963	80.03 K	787
	18-39	12,533	28.53 K	440
	40-69	34,190	36.94 K	926
	>=70	16,227	14.58 K	1 113
Regional	All ages	6,402	3.63 K	1762
	18-39	1,933	1.31 K	1 477
	40-69	3,384	1.65 K	2 056
	>=70	1,085	0.68 K	1 598

PCC: Possible chronic cough.

Supportive Supplementary data

Supplementary figures

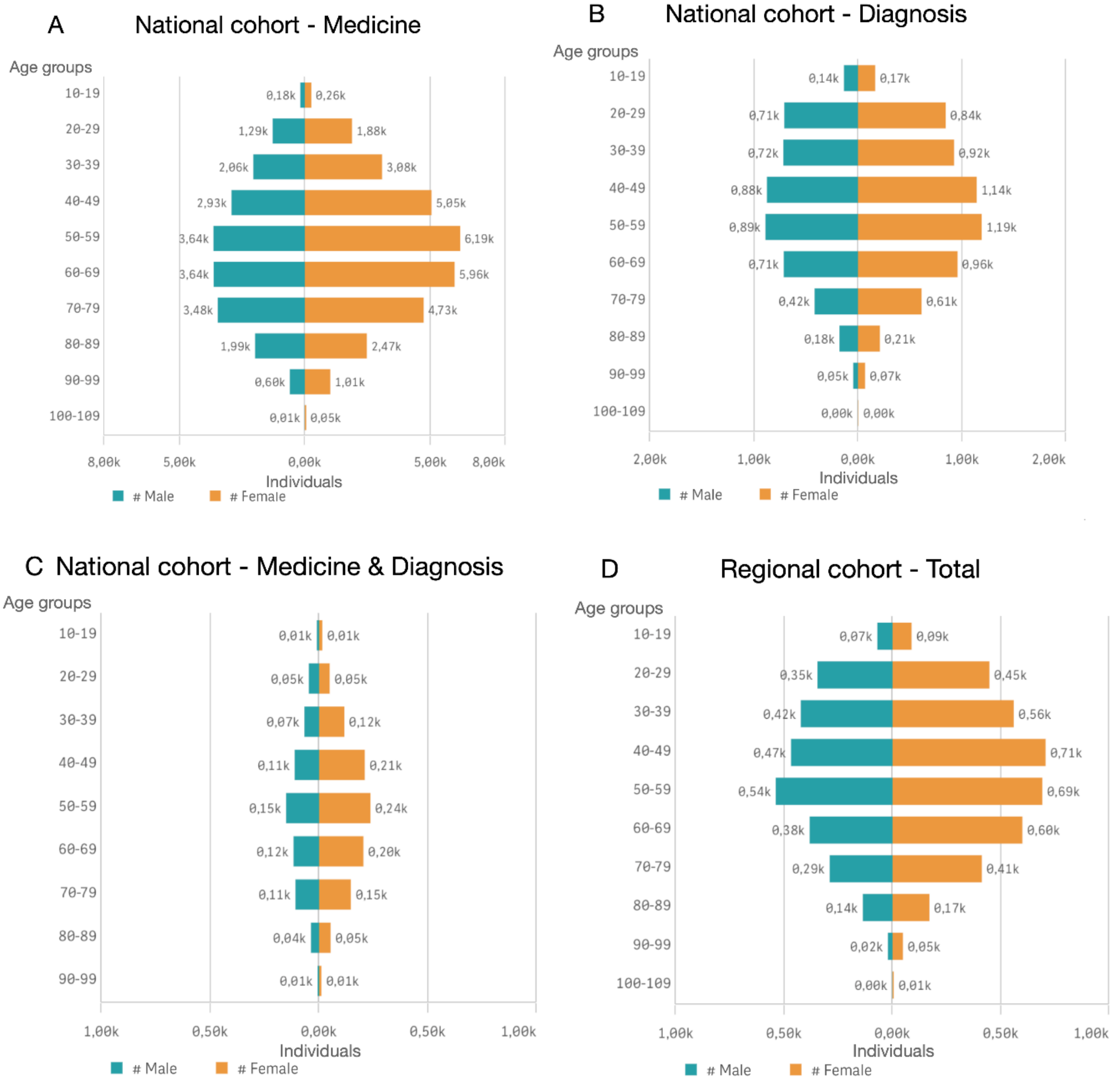

Figure S1. Age and gender distribution of individuals with possible chronic cough, by A) those identified from prescriptions only, B) those identified from diagnosis only, C) those identified with both prescriptions and diagnosis, D) those identified in the regional cohort.

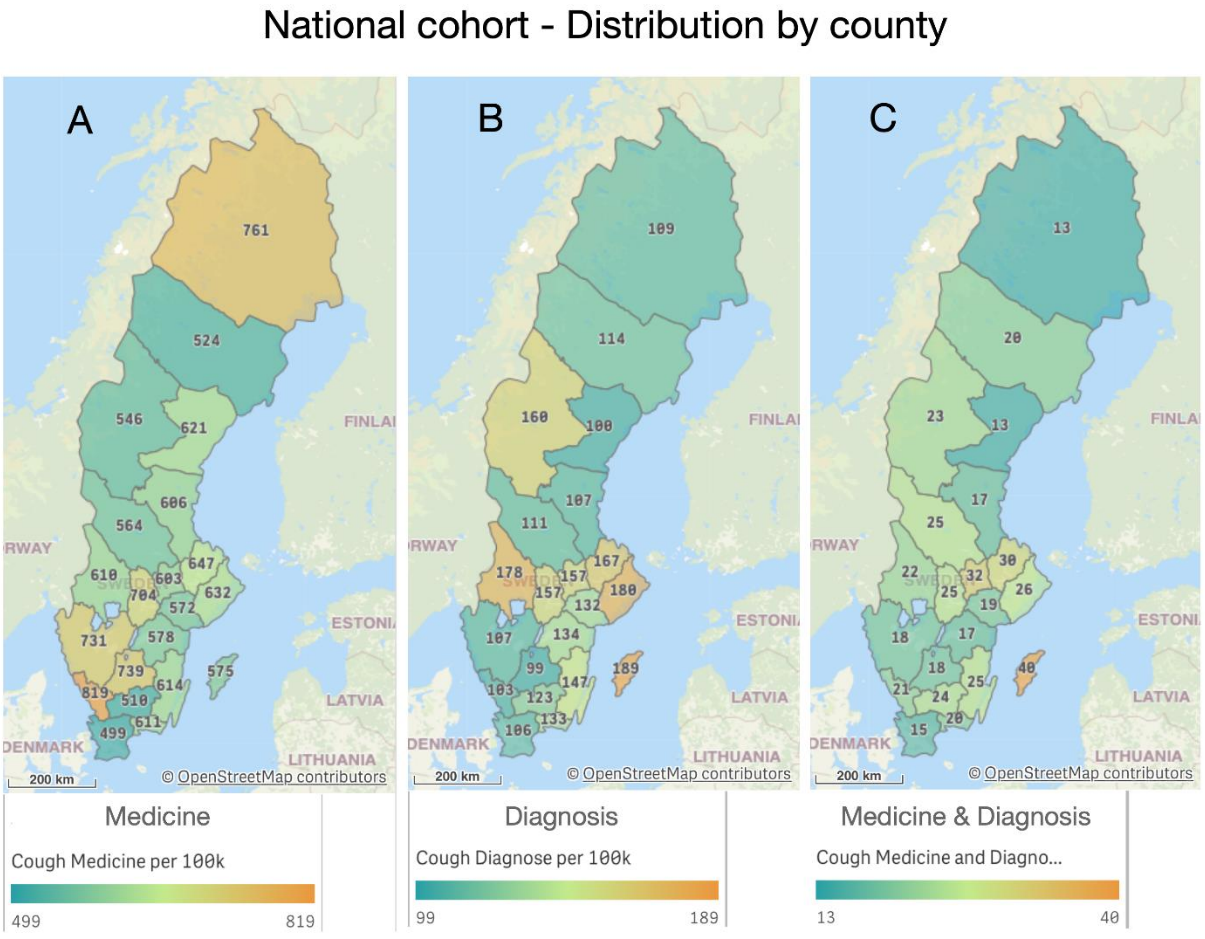

Figure S2. Distribution in the 21 county councils in Sweden of included individuals with chronic cough due to ICD 10 code R05 or frequent dispensed prescriptions of relevant cough medicine

Supplementary tables

Table S1. Number of individuals with possible chronic cough stratified by type of cough medicine and number of dispenses per cough medicines 2016-2018.

Group	ATC	Name	1 DISP	2-3 DISP	4-6 DISP	>6 DISP	Total
Exp	R05CB01	Acetylcysteine	6,440<br>20.7 %	12,090<br>38.9 %	7,220<br>23.2 %	5,301<br>17.1 %	31,051<br>100.0 %
Opiates	R05DA04	Codeine	314<br>17.2 %	484<br>26.5 %	325<br>17.8 %	705<br>38.6 %	1,828<br>100.0 %
CS/ Exp	R05CB02	Bromhexine	225<br>40.8 %	131<br>23.7 %	75<br>13.6 %	121<br>21.9 %	552<br>100.0 %
CS/ Exp	R05FB02	Cough Suppressants and expectorants	162<br>47.5 %	122<br>35.8 %	44<br>12.9 %	13<br>3.8 %	341<br>100.0 %
CS	R05DA07	Noscapine	220<br>80.6 %	48<br>17.6 %	4<br>1.5 %	1<br>0.4 %	273<br>100.0 %
CS	R05DA20	Combinations	1,269<br>49.8 %	964<br>37.8 %	255<br>10.0 %	60<br>2.4 %	2,548<br>100.0 %

CS	R05FA02	Opium derivatives and expectorants	8,868<br>25.8 %	19,224<br>56.0 %	5,163<br>15.0 %	1,074<br>3.1 %	34,329<br>100.0 %
Anti-E	R06AA02	Dimenhydrinate	7<br>36.8 %	9<br>47.4 %	3<br>15.8 %	0<br>0.0 %	19<br>100.0 %
**Total**			14,841<br>26.6 %	31,454<br>56.3 %	12,976<br>23.2 %	7,206<br>12.9 %	55,823<br>100.0 %

One patient may have multiple medicines and be counted on multiple rows. Exp: Expectorants, CS: Cough Suppressant, Anti-E: Anti-emetic.

Table S2. Individuals identified with cough in the national cohort (25, 046) and the regional cohort (11,647). Stratification of excluded patients.

Group	Time	Code	Excluded by		Total
			National cohort	Regional cohort	
ACEi	Anytime	ATC: C09	106,675	2,533	109,208
Antibiotics	+/- 8w from ID	ATC: J01	140,250	2,331	142,637
Cancer	Anytime	ICD: C32, C33 and C34	4,374	39	4 413
Acute cough	+/- 8w from ID	ICD: J06, J20, J21 and J22	10,255	2,027	12,599
Pneumonia	+/- 8w from ID	ICD: J15, J16, J17 and J18	21,063	728	21,804
TBC	Anytime	ICD: A15, A16, A18 and A19	256	6	262
Emergency visit for R05	Anytime	ICD: R05	1,532	0	1,532
Other lung diseases	Anytime	ICD: J84	3,191	35	3,227
COPD	Anytime	ICD: J44	32,217	462	32,707
**Total**			193,083	5,249	198,697

One patient might have multiple exclusion criteria fulfilled for the same exclusion step and thus be counted on multiple rows.

Table S3. Description of the ICD-10 and ATC codes used in exclusion.

Exclusion group	ATC/ICD	Description
ACEi	ATC C09	Agents acting on the Renin-Angiotensin system
Antibiotics	ATC J01	Anti-bacterials for systemic use
Cancer	C32	Malignant Neoplasm Of Larynx
	C33	Malignant Neoplasm Of Trachea and Bronchus
	C34	Malignant Neoplasm Of Lung
Acute cough	J06	Acute Upper Respiratory Infections Of Multiple And Unspecified Sites
	J21	Acute Bronchiolitis
	J22	Unspecified Acute Lower Respiratory Infection
Pneumonia	J15	Bacterial Pneumonia, Not Elsewhere Classified
	J16	Pneumonia Due To Other Infectious Organisms, Not Elsewhere Classified
	J17	Pneumonia In Diseases Classified Elsewhere
	J18	Pneumonia, Unspecified Organism
Tuberculosis	A15	Respiratory Tuberculosis

	A16	Respiratory Tuberculosis, Not Confirmed Bacteriologically Or Histologically
	A18	Tuberculosis Of Other Organs
	A19	Miliary Tuberculosis
Emergency Trtmnt	R05	Acute or Emergency treatment of cough
Other lung diseases	J84	Other Interstitial Pulmonary Diseases
COPD	J44	Other Chronic Obstructive Pulmonary Disease

Table S4. Diagnoses used for RCC classification and number of individuals per group.

Co-diagnosis at R05 visit 2016-2018		
**Diagnosis**	**Individuals**	**%**
R06 Abnormalities of Breathing	737	20.7
J30 Vasomotor and Allergic Rhinitis	413	11.6
R50 Fever of Other and Unknown Origin	374	10.5
J45 Asthma	369	10.4
R07 Pain in Throat and Chest	285	8.0
K21 Gastro-Esophageal Reflux Disease	246	6.9
R49 Voice and Resonance Disorders	182	5.1
R53 Malaise and Fatigue	137	3.8
R13 Aphagia and Dysphagia	93	2.6
R04 Hemorrhage from Respiratory Passages	91	2.6
J31 Chronic Rhinitis, Nasopharyngitis and Pharyngitis	89	2.5
J34 Other and Unspecified Disorders of Nose and Nasal Sinuses	70	2.0
R10 Abdominal and Pelvic Pain	68	1.9
R51 Headache	59	1.7
I48 Atrial Fibrillation and Flutter	58	1.6
R52 Pain, Not Elsewhere Classified	51	1.4
R11 Nausea and Vomiting	50	1.4
J38 Diseases of Vocal Cords and Larynx, Not Elsewhere Classified	44	1.2
G47 Sleep Disorders	43	1.2
J47 Bronchiectasis	39	1.1
R55 Syncope and Collapse	32	0.9
F45 Somatoform Disorders	31	0.9
R00 Abnormalities of Heartbeat	30	0.8
J33 Nasal Polyp	29	0.8
R12 Heartburn	29	0.8
F41 Other Anxiety Disorders	28	0.8
R63 Symptoms and Signs Concerning Food and Fluid Intake	25	0.7
J39 Other Diseases of Upper Respiratory Tract	24	0.7

R09 Other Symptoms and Signs Involving the Circulatory and Respiratory System	24	0.7
R42 Dizziness and Giddiness	23	0.7

One individual might be counted on multiple rows.

Table S5. Number of individuals with dispense of medicines from ATC groups R01, R03 and A02.

Individuals with PCC (n=62 963)	Example of use	Medicine retrieved 2016-2018	Medicine retrieved 2006-2018
R01 Drugs for Nasal conditions	Nasal conditions and allergic rhinitis	12,764	22,707
R03 Drugs for Obstructive Pulmonary disease	Asthma + COPD	22,152	23,143
A02 Drugs for acid related disorders	GERD	11,858	17,304
**Total**		26,632	31,969

One patient may be counted on multiple rows.

Table S6. Co-diagnosis at R05 visits 2016-2018.

Co-diagnosis at R05 visit 2016-2018		
**Diagnosis**	**Individuals**	**%**
R06 Abnormalities of Breathing	737	20.7
J30 Vasomotor and Allergic Rhinitis	413	11.6
R50 Fever of Other and Unknown Origin	374	10.5
J45 Asthma	369	10.4
R07 Pain in Throat and Chest	285	8.0
K21 Gastro-Esophageal Reflux Disease	246	6.9
R49 Voice and Resonance Disorders	182	5.1
R53 Malaise and Fatigue	137	3.8
R13 Aphagia and Dysphagia	93	2.6
R04 Hemorrhage from Respiratory Passages	91	2.6
J31 Chronic Rhinitis, Nasopharyngitis and Pharyngitis	89	2.5
J34 Other and Unspecified Disorders of Nose and Nasal Sinuses	70	2.0
R10 Abdominal and Pelvic Pain	68	1.9
R51 Headache	59	1.7
I48 Atrial Fibrillation and Flutter	58	1.6
R52 Pain, Not Elsewhere Classified	51	1.4
R11 Nausea and Vomiting	50	1.4
J38 Diseases of Vocal Cords and Larynx, Not Elsewhere Classified	44	1.2

G47 Sleep Disorders	43	1.2
J47 Bronchiectasis	39	1.1
R55 Syncope and Collapse	32	0.9
F45 Somatoform Disorders	31	0.9
R00 Abnormalities of Heartbeat	30	0.8
J33 Nasal Polyp	29	0.8
R12 Heartburn	29	0.8
F41 Other Anxiety Disorders	28	0.8
R63 Symptoms and Signs Concerning Food and Fluid Intake	25	0.7
J39 Other Diseases of Upper Respiratory Tract	24	0.7
R09 Other Symptoms and Signs Involving the Circulatory and Respiratory System	24	0.7
R42 Dizziness and Giddiness	23	0.7

One individual might be counted on multiple rows.

Table S7. Clinic type that sets the cough diagnosis and Clinic type of Cough Medicine prescriber.

**R05 diagnosis recordings**		**Cough medicine prescriptions**	
**Clinic type**	**%**	**Clinic type**	**%**
Internal medicine	29.5	Primary care	82.5
Ear, nose and throat	19.9	Unknown	3.5
Lung	15.4	Internal medicine	3.0
Emergency	12.0	Ear, nose and throat	1.5
Allergy	7.7	Occupational healthcare	1.4
Infectious disease	5.2	Surgical	0.9
Others	10.3	Others	7.2
Total	100.0	Total	100.0

Table S8. List of medicines dispensed by the individuals in the national cohort 2016-2018.

**ATC**	**# Individuals**	**% Individuals**	**# Expeditions**
R05 Medicines against cough and common cold*	57,171	90.8	266,528
R03 Anti-obstructive respiratory medicines	27,301	43.4	285,116
A02 Medicines for acidity-related symptoms	23,300	37.0	209,155
J01 Anti-bacterials for systemic use	22,504	35.7	43,880
R06 Antihistaminic drugs for systemic use	17,014	27.0	99,259
R01 Medicines for nasal conditions	16,787	26.7	64,547
H02 Corticosteroids for systemic use	14,127	22.4	55,403

N03 Antiepileptics	5,512	8.6	121,892
J05 Antivirals for systemic use	3,590	5.7	15,050
J02 Antimycotic agents for systemic use	2,495	4.0	5,692
J07 Vaccines	852	1.4	1,235
R02 Medicines for throat and pharynx	68	0.1	78

*R05 is included in the inclusion criteria, outlined in Table S3.

One patient might be counted on multiple rows.

Table S9. Number of individuals with possible chronic cough stratified by type of cough medicine and number of dispenses per cough medicines 2016-2018.

Group	ATC	Name	1 DISP	2-3 DISP	4-6 DISP	>6 DISP	Total
Exp	R05CB01	Acetylcysteine	6,440<br>20.7 %	12,090<br>38.9 %	7,220<br>23.2 %	5,301<br>17.1 %	31,051<br>100.0 %
Opiates	R05DA04	Codeine	314<br>17.2 %	484<br>26.5 %	325<br>17.8 %	705<br>38.6 %	1,828<br>100.0 %
CS/ Exp	R05CB02	Bromhexine	225<br>40.8 %	131<br>23.7 %	75<br>13.6 %	121<br>21.9 %	552<br>100.0 %
CS/ Exp	R05FB02	Cough Suppressants and expectorants	162<br>47.5 %	122<br>35.8 %	44<br>12.9 %	13<br>3.8 %	341<br>100.0 %
CS	R05DA07	Noscapine	220<br>80.6 %	48<br>17.6 %	4<br>1.5 %	1<br>0.4 %	273<br>100.0 %
CS	R05DA20	Combinations	1,269<br>49.8 %	964<br>37.8 %	255<br>10.0 %	60<br>2.4 %	2,548<br>100.0 %
CS	R05FA02	Opium derivatives and expectorants	8,868<br>25.8 %	19,224<br>56.0 %	5,163<br>15.0 %	1,074<br>3.1 %	34,329<br>100.0 %
Anti-E	R06AA02	Dimenhydrinate	7<br>36.8 %	9<br>47.4 %	3<br>15.8 %	0<br>0.0 %	19<br>100.0 %
**Total**			14,841<br>26.6 %	31,454<br>56.3 %	12,976<br>23.2 %	7,206<br>12.9 %	55,823<br>100.0 %

One patient may have multiple medicines and be counted on multiple rows. Exp: Expectorants, CS: Cough Suppressant, Anti-E: Anti-emetic.

Click here to access/download;Figure;Fig1_Dataset_creation.pdf

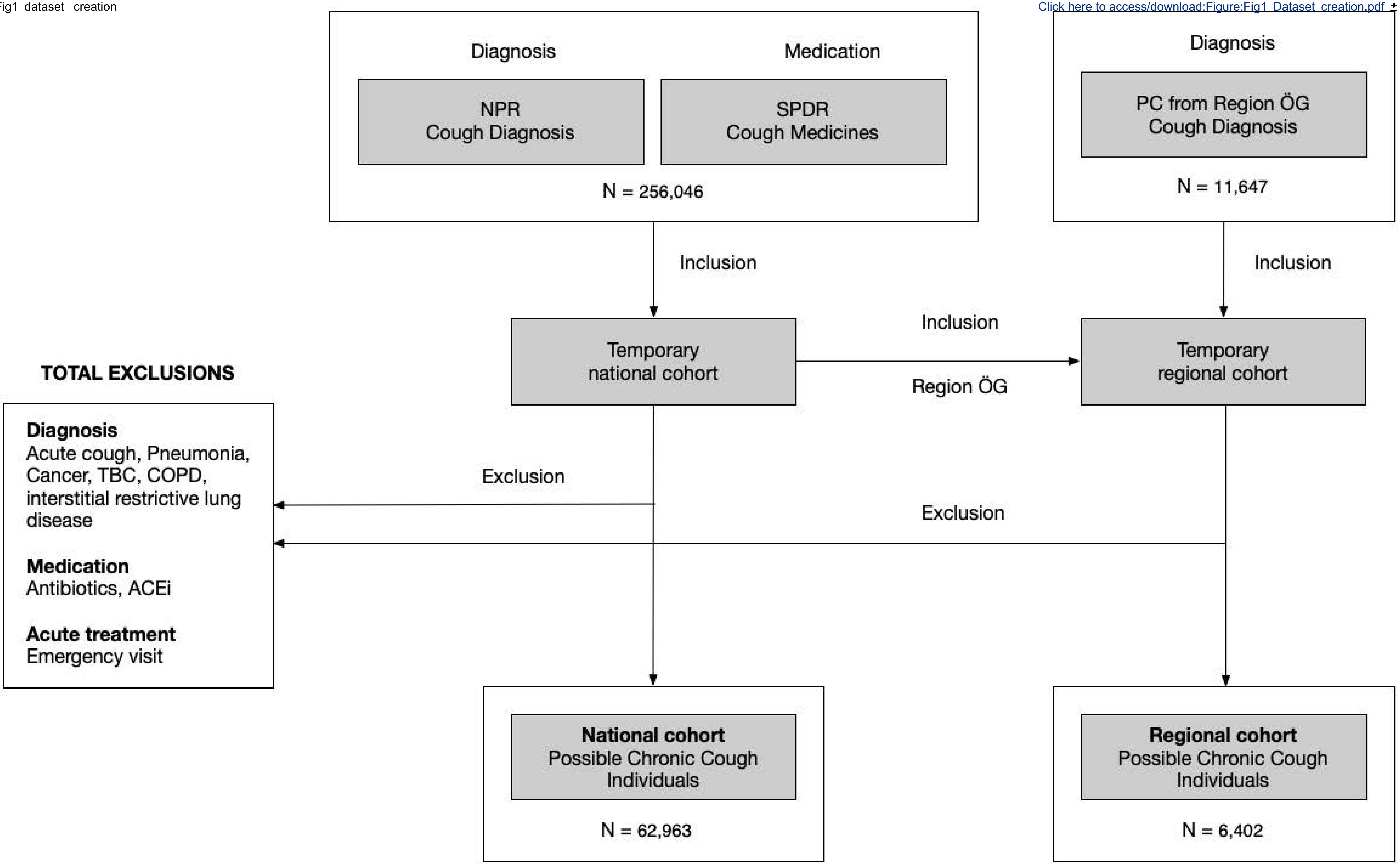

Fig2AB

Click here to access/download;Figure;Fig2AB.tif

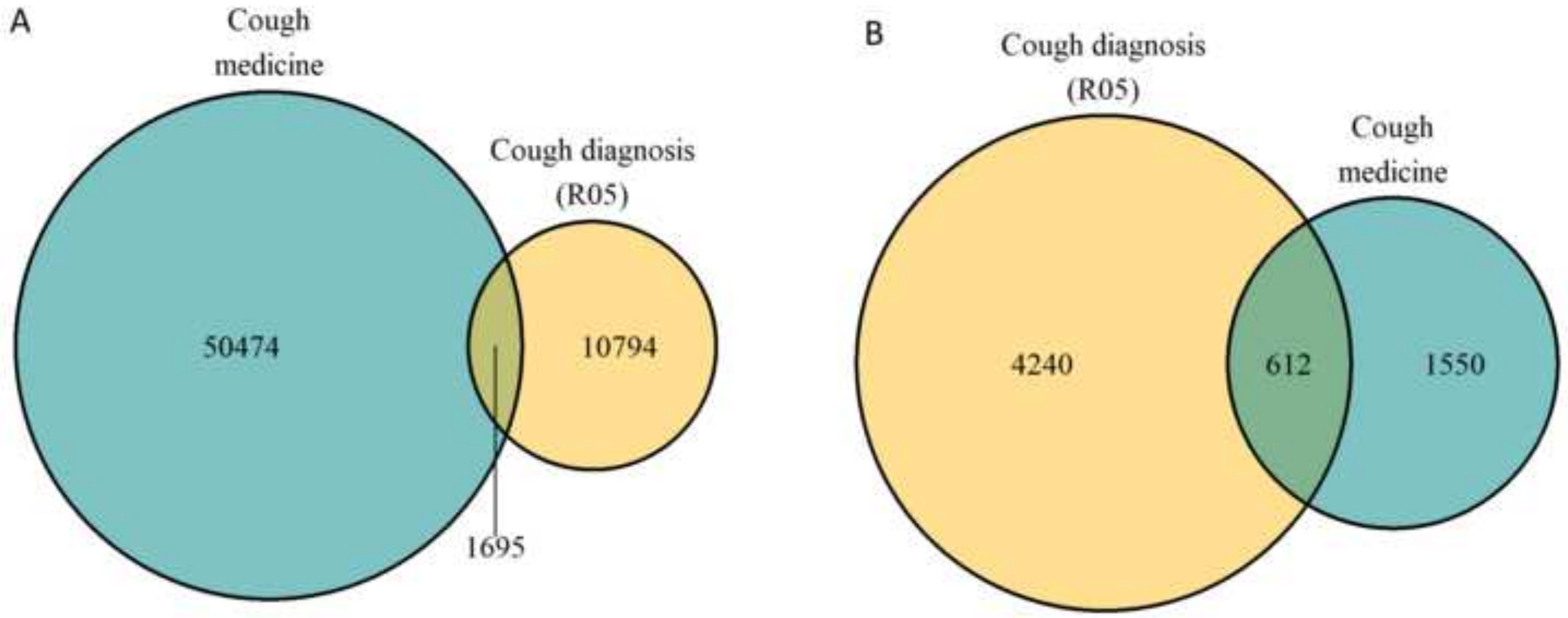

Fig3_Age_Gender_National

Click here to access/download;Figure;Fig3_Age_Gender_National.tiff

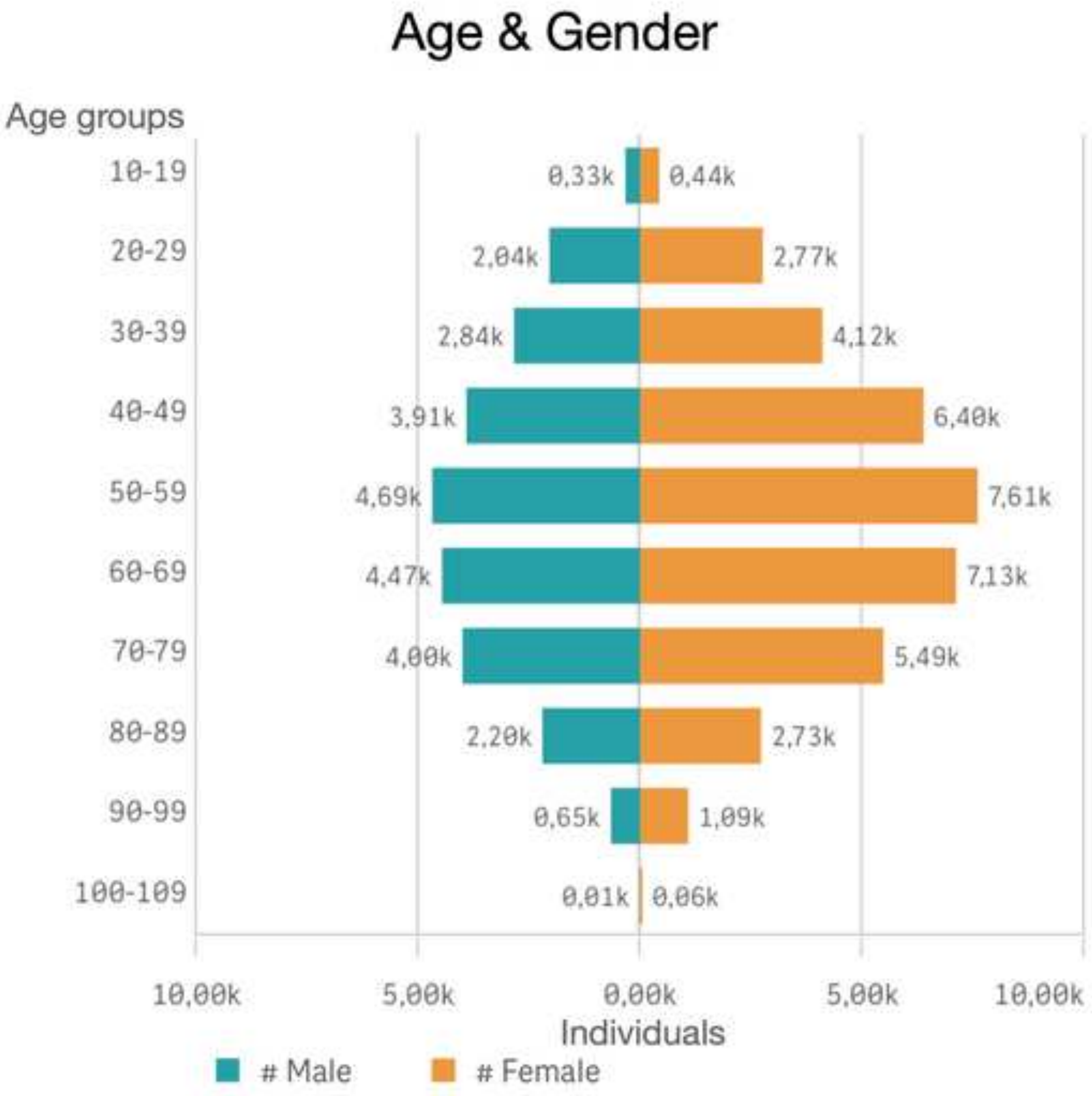

Click here to
access/download;Figure;Fig4_Freq_visits_medicine.tiff

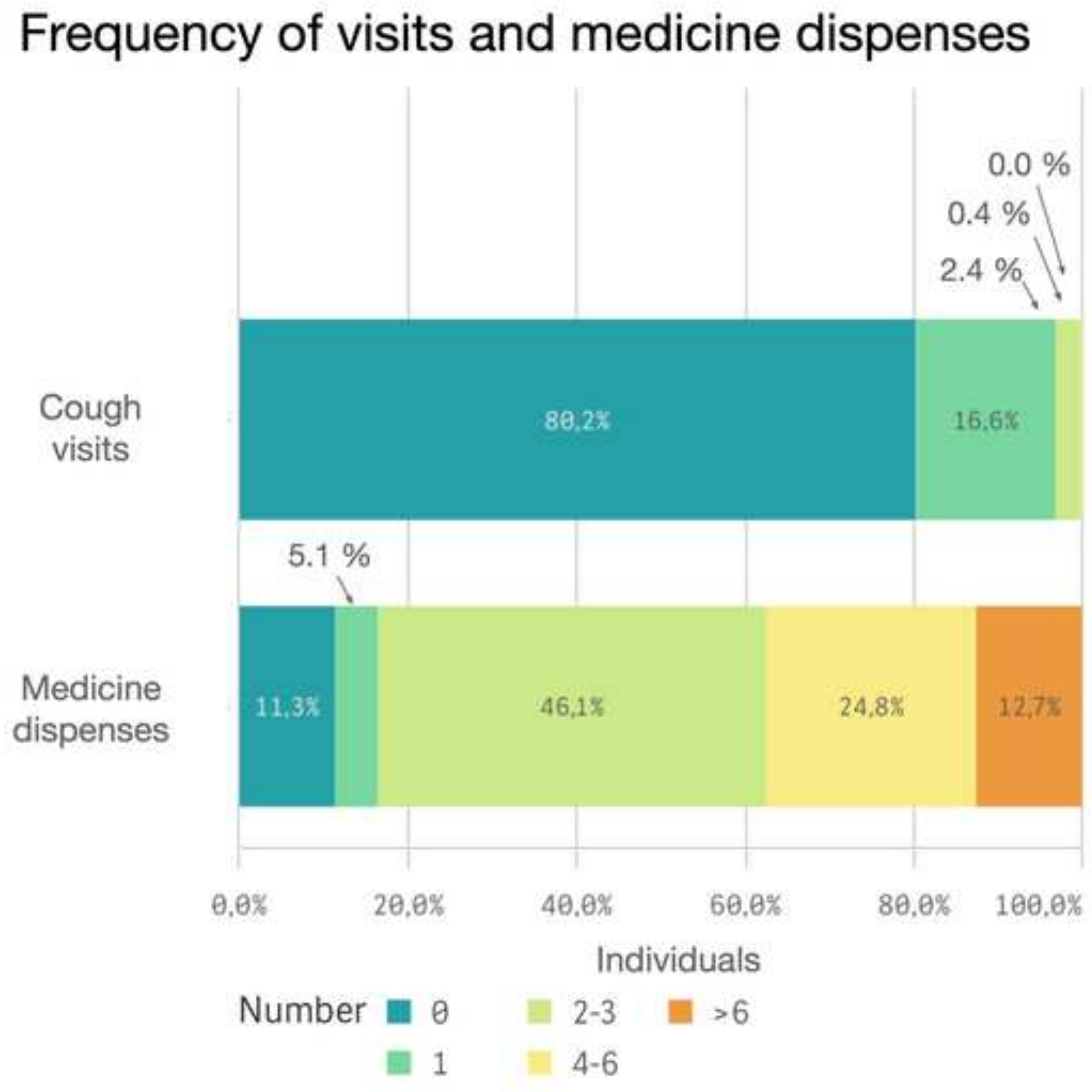

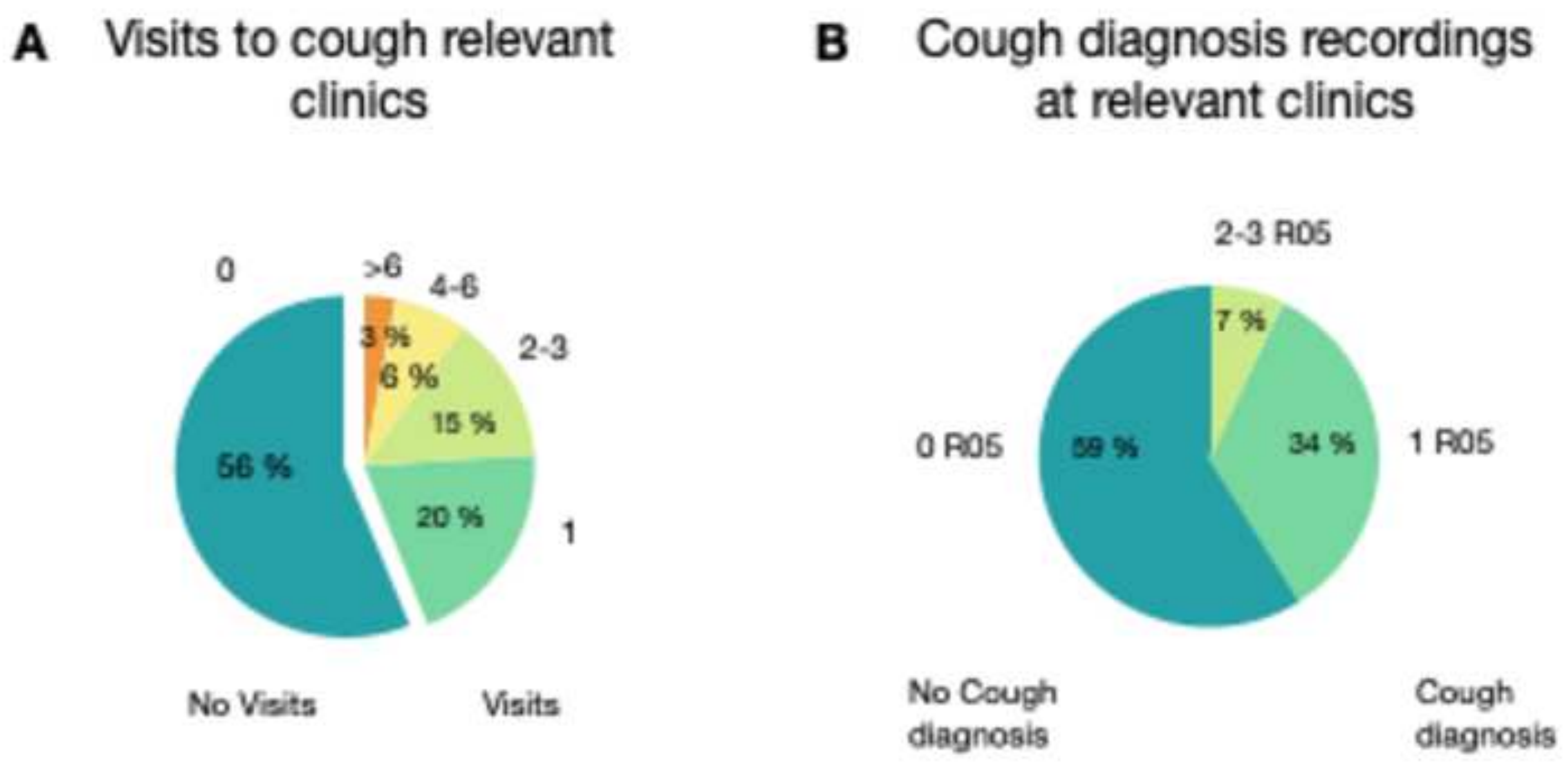
A
Visits to cough relevant clinics
0
>6
4-6
2-3
1
3 %
6 %
15 %
56 %
20 %
No Visits
Visits
B
Cough diagnosis recordings at relevant clinics
2-3 R05
7 %
0 R05
59 %
34 %
1 R05
No Cough diagnosis
Cough diagnosis

Click here to access/download;Figure;Fig6AB.pdf

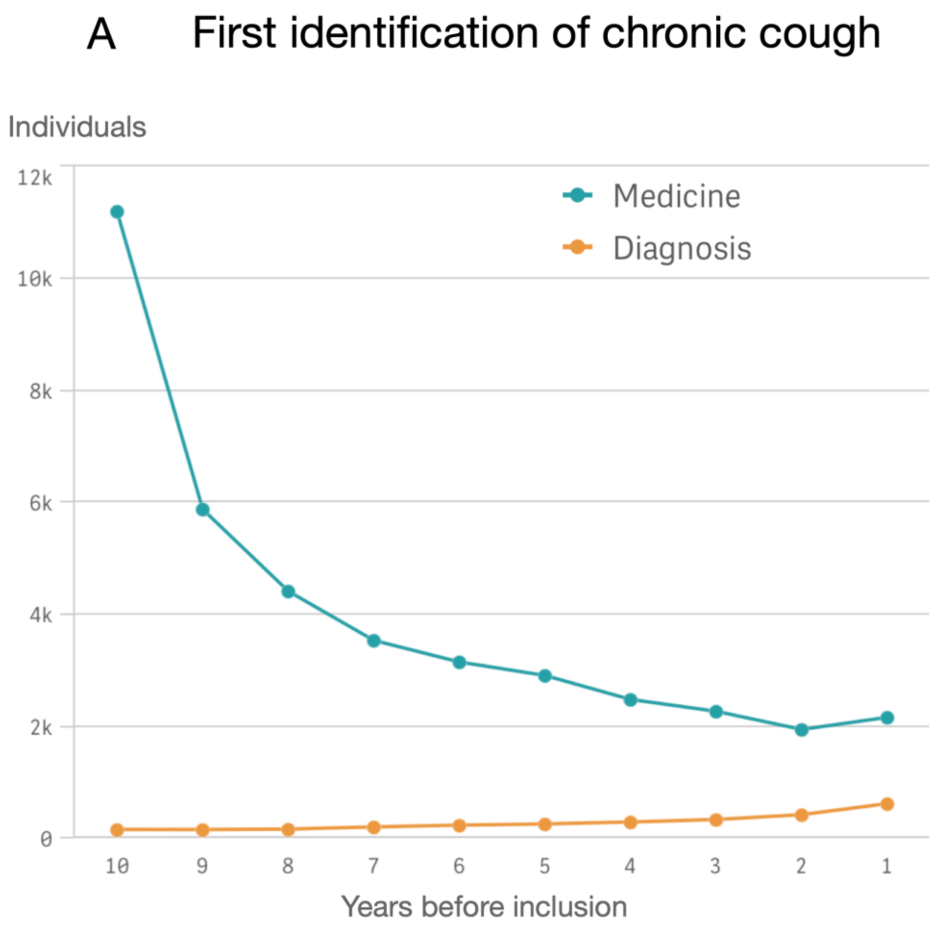

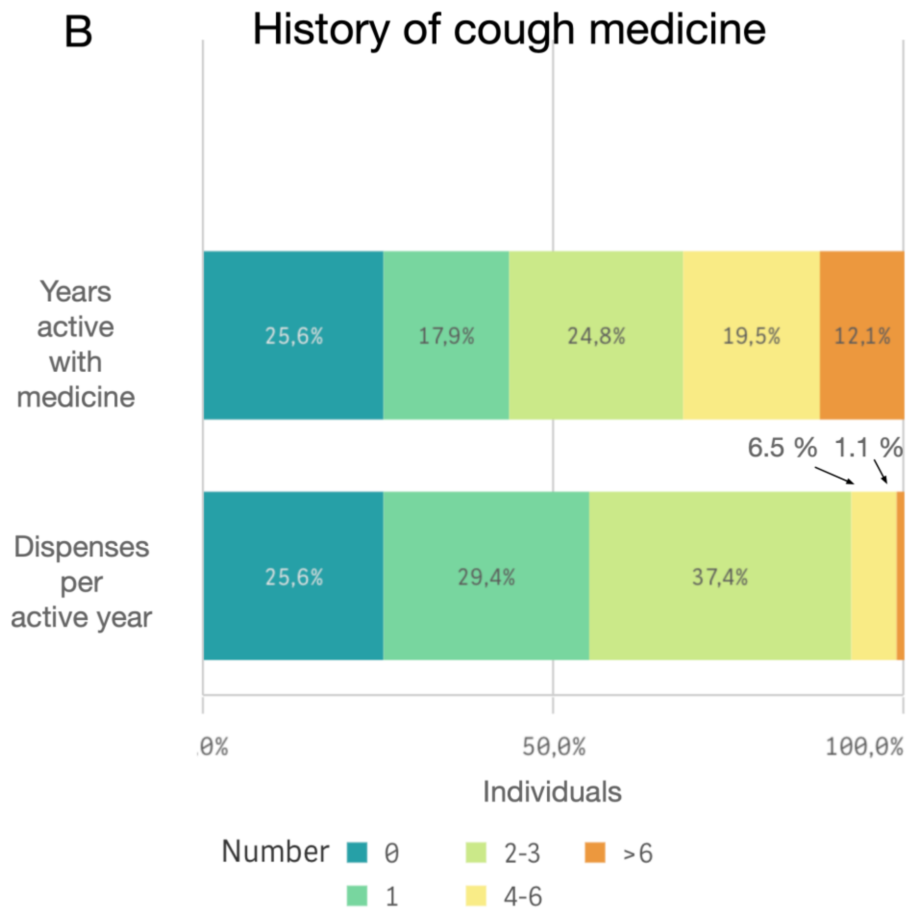

Suplementary

Click here to access/download
Supporting Information
Suppl cough register study - Plos One.docx
