# Supplementary material for: Characteristics, demographics, and epidemiology of possible chronic cough in Sweden: A nationwide register-based cohort study
Source: PLoS One. 2024 Jul 24;19(7):e0303804. doi: 10.1371/journal.pone.0303804 (PMC11268580; doi:10.1371/journal.pone.0303804)
Supplement: Attachment — Submitted filename: Response to Reviewers .docx [file pone.0303804.s005.docx]

**Manuscript ID:** PONE-D-23-39518

**Manuscript Title:** Characteristics, demographics, and epidemiology of possible chronic cough in Sweden: A nationwide register-based cohort study

## Response to reviewers’ comments

**Authors’ general reply:** We thank for the opportunity to revise and improve our manuscript, based on the reviewers’ comments. We feel the manuscript has improved significantly after implementing these changes, and hope that it now fulfils the journal’s requirements for publication. Otherwise, we are happy to make further amendments as required.

*Reviewers’ comments:*

*Reviewer #1: Dear Author
The register-based cohort study in a Sweden population is very interesting.*

**Reply:** Thank you for this positive comment!

*But the data is only about demographics of population with cough.
There is no new or interesting finding related to cough in population in the manuscript.*

**Reply:** This is an important comment. The cohort described shows a cohort that in most aspects is similar to those found in clinical studies on chronic cough. This is important to show, as this is the first study identifying patients with chronic cough using the nationwide Swedish healthcare register data, indicating that our method finds patients with chronic cough similar to those identified in clinical studies. In that aspect, the findings are novel. We have now emphasized this in the discussion section, line 304-306, by adding the following: “This indicates that the patient group identified with PCC using the Swedish healthcare register data is comparable to chronic cough cohorts recruited in clinical studies.”

Also, the real-world prevalence within the healthcare setting, as well as where the patients are treated, has not been shown before.

*Reviewer #2: A good study addressing chronic cough.
\# Smoking is also one of the major causes for chronic cough even without established COPD. Addressing it becomes mandatory.*

**Reply:** This is an important comment. Unfortunately, the Swedish healthcare register data does not include any data on smoking, and therefore this important factor could not be included in the analysis. This is addressed in the discussion, section on limitations, but we have now also added this to the methods section, lines 145-146: “As the registers do not have information on smoking, smoking status could not be used in the exclusion criteria.”

*# Not only treating but also assessing the treatment response will help physicians across world to provide better patient care. Provide the data for treatment response if available.*

**Reply:** Unfortunately, treatment response is also not included in the Swedish healthcare register data, and could therefore not be included. This is now highlighted in the section on limitations, lines 347-349: “Also, treatment response is not included in the registers, and therefore treatment response could not be assessed.”

*# Discussion seems superficial. Dig and include more comparative studies.*

**Reply:** Thank you for this suggestion. We have now made some adjustments: In response to a comment made by reviewer 1, we have emphasized the novelty of the method used (see reply above). Also, we have added a deeper interpretation of our finding regarding patients being largely treated in primary care only by adding the following in lines 272-274: “A recent international Delphi study identified variability between geographical regions regarding the treatment of chronic cough, and a need for increased access to specialist care to improve patient care.” with reference to “Song WJ et al, 2023, ERJ Open Research”. Finally, we added to the discussion on opioid use in chronic cough, lines 312-313: “This is a somewhat higher proportion of opioid use compared with recent data from a study in Florida.” with reference to “Yang S et al, 2023, J Clin Med”.
